# A self-assembled nanophotosensitizer targets lysosomes and induces lysosomal membrane permeabilization to enhance photodynamic therapy†

**DOI:** 10.1039/d3sc00455d

**Published:** 2023-04-19

**Authors:** Youyou Li, Wenbo Han, Deyan Gong, Taokun Luo, Yingjie Fan, Jianming Mao, Wenwu Qin, Wenbin Lin

**Affiliations:** a Department of Chemistry, The University of Chicago Chicago Illinois 60637 USA wenbinlin@uchicago.edu; b Department of Radiation and Cellular Oncology and Ludwig Center for Metastasis Research, The University of Chicago Chicago IL 60637 USA; c Key Laboratory of Nonferrous Metal Chemistry and Resources Utilization of Gansu Province and State Key Laboratory of Applied Organic Chemistry, College of Chemistry and Chemical Engineering, Lanzhou University Lanzhou 730000 China

## Abstract

We report the self-assembly of amphiphilic BDQ photosensitizers into lysosome-targeting nanophotosensitizer BDQ-NP for highly effective photodynamic therapy (PDT). Molecular dynamics simulation, live cell imaging, and subcellular colocalization studies showed that BDQ strongly incorporated into lysosome lipid bilayers to cause continuous lysosomal membrane permeabilization. Upon light irradiation, the BDQ-NP generated a high level of reactive oxygen species to disrupt lysosomal and mitochondrial functions, leading to exceptionally high cytotoxicity. The intravenously injected BDQ-NP accumulated in tumours to achieve excellent PDT efficacy on subcutaneous colorectal and orthotopic breast tumor models without causing systemic toxicity. BDQ-NP-mediated PDT also prevented metastasis of breast tumors to the lungs. This work shows that self-assembled nanoparticles from amphiphilic and organelle-specific photosensitizers provide an excellent strategy to enhance PDT.

## Abstract 2

Self-assembly of amphiphilic photosensitizer BDQ into BDQ-NP enables stable incorporation of BDQ into lysosome membranes to cause continuous lysosomal membrane permeabilization for highly effective photodynamic therapy.

## Graphical abstract

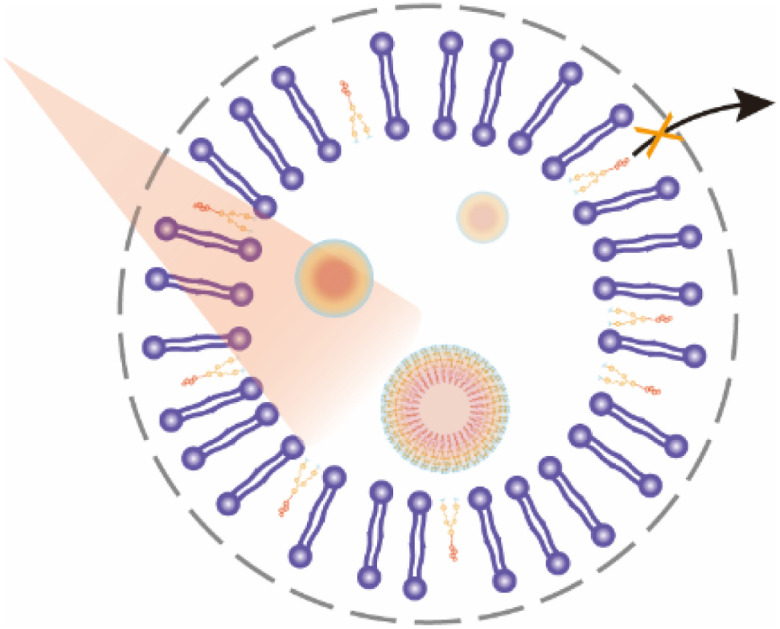

## Introduction

Photodynamic therapy (PDT) elicits cytotoxicity by damaging vital biomolecules and organelles with reactive oxygen species (ROS) generated from a combination of light and photosensitizers (PSs).^1,2^ PSs can target different milieus of tumours, such as stroma,^3,4^ cellular receptors,^5^ and subcellular organelles,^6–12^ to provide additional strategies for enhancing their antitumor effects.^13–16^ Some amphiphilic PSs have shown organelle-specific accumulation and provide tumour-targeted PDT.^16–21^

As acidic organelles, lysosomes play vital roles in regulating cell homeostasis by digesting the taken-up extracellular materials.^22–25^ Disruption of lysosomal functions *via* lysosomal membrane permeabilization (LMP) can cause lysosome-dependent cell death.^26^ LMP activates effectors such as ROS, Bax, and iron, leading to cell apoptosis, pyroptosis,^27^ and ferroptosis.^28,29^ Several lysosome-targeting PSs were studied as anticancer LMP inducers in recent years. Ir^III^ complexes,^30^ Ru^II^ complexes,^30–32^ and some organic PSs^33,34^ showed lysosomal localization and PDT *in vitro*; a few of them were examined for PDT *in vivo via* intratumoral injection, leading to moderate antitumor efficacy.^30^ Existing lysosome-targeting PSs leak from lysosomes and redistribute to the cytoplasm during PDT-induced LMP. Cancer cells can recover from this kind of lysosomal disruption by reducing lysosome exposure to PSs and hence photochemical damage.^35^ The PDT efficacy can be substantially enhanced if lysosome-targeting PSs do not leak from lysosomes during light irradiation, thus causing continuous LMP and irreparable damage to cancer cells.

Boron-dipyrromethene (BOD) derivatives have been extensively explored as PSs for PDT owing to their high molar extinction coefficients, low dark toxicity, resistance to photobleaching, and high chemical stability.^36–43^ BOD derivatives can also be systematically modified through organic synthesis to enhance their photophysical and pharmacokinetic properties and PDT efficacy. For example, heavy atom substitution of BOD can significantly increase single oxygen (^1^O_2_) yield by enhancing intersystem crossing *via* spin–orbit coupling,^44,45^ while enlargement of conjugation systems can red-shift the absorption toward the near-infrared region with deeper tissue penetration.^46,47^

We have recently shown the noncovalent insertion of cholesterol-based prodrugs into phospholipid bilayers for drug delivery.^48,49^ It is hypothesized that cholesterol could act as an anchor to insert cholesterol-linked PSs into lipid bilayers of lysosomes. Cholesterol modification of charged BOD derivatives provides hydrophobic ends to form amphiphiles which can self-assemble into micelles and other types of nanostructures.^50^ Additionally, cholesterol conjugation can further modify the structure of BOD derivatives to reduce pi–pi stacking interactions between the large conjugated systems, which reduces aggregation-induced quenching of photo-excited PSs.

**Scheme 1:**
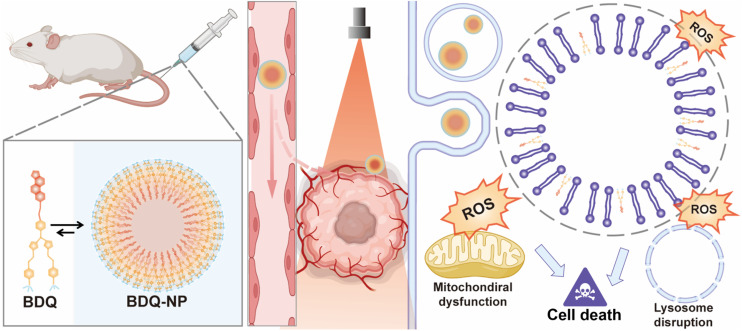
Schematic showing enhanced PDT *via* lysosome targeting and LMP. The BDQ-NP enhances PDT efficacy by (1) self-assembly into BDQ-NP to prolong blood circulation and increase accumulation in tumors; (2) stable BDQ incorporation into lysosome membranes to cause continuous LMP under light irradiation, leading to irreparable lysosomal disruption; (3) generation of large quantities of ROS and downstream organelle dysfunction to induce cell death.

Herein, we report the design and synthesis of a novel BOD-based, lysosome-targeting, self-assembled nanophotosensitizer BDQ-NP (Scheme 1). Amphiphilic BDQ was synthesized from BOD in four steps and self-assembled into BDQ-NP particles in aqueous solution. Molecular dynamics (MD) simulation and live cell imaging showed that BDQ inserted into lysosome membranes and caused LMP upon light irradiation. As a result, BDQ efficiently generated ROS and displayed nearly two orders of magnitude greater cytotoxicity than non-lysosome-targeting BDS. With good accumulation in tumours, the intravenously administered BDQ-NP plus light irradiation showed strong antitumor efficacy on subcutaneous CT26 colorectal and orthotopic 4T1 breast tumour models and efficiently prevented lung metastasis of 4T1 tumours.

## Results and discussion

### Design and synthesis of the BDQ-NP

**Fig. 1:**
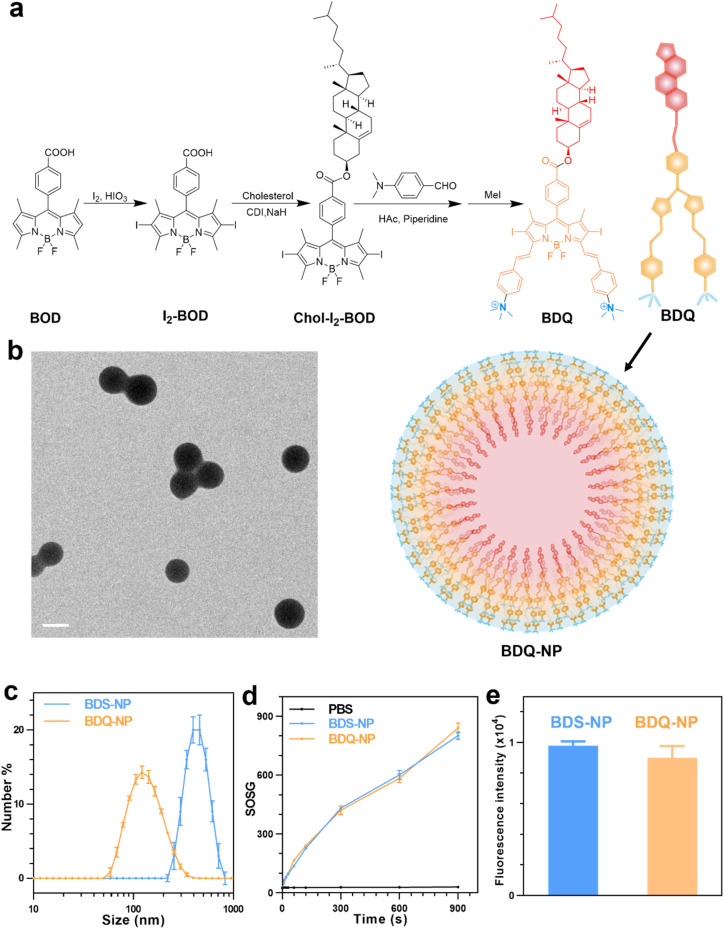
(a) Synthesis of BDQ and its self-assembly into BDQ-NP in aqueous solution. (b) TEM image of the BDQ-NP. Scale bar = 100 nm. (c) Number-average sizes of the BDS-NP and BDQ-NP in PBS measured by DLS. (d) SOSG fluorescence signals of the BDQ-NP and BDS-NP in PBS solution with 0.5% (V/V) Triton-X-100 upon irradiation at 660 nm (*n* = 3). (e) Cellular concentrations of the BDS-NP and BDQ-NP by flow cytometry analysis after 8 h incubation.

BOD was first iodinated and then coupled to cholesterol to afford Chol-I_2_-BOD. Treatment of Chol-I_2_-BOD with benzaldehyde or 4-*N*,*N*-dimethylaminobenzaldehyde followed by quaternization with methyl iodide produced two new BOD-based PSs BDS or BDQ, respectively (Fig. 1a). BDS and BDQ showed similar UV-vis spectra (Fig. S14†) and displayed strong fluorescence at ∼660 nm (Fig. S15†).

The critical micelle concentration (CMC) of BDQ was determined to be 8.3 μM (Fig. S17†), which supports efficient and stable formulation of micelle-like nanostructures. The addition of 1 mL of 0.28 mM BDQ in dimethyl sulfoxide (DMSO) to 17 mL phosphate-buffered saline (PBS) afforded self-assembled BDQ-NP nanoparticles, with an average size of 128.9 nm (Fig. 1c) and a polydispersity index (PDI) of 0.19 (Fig. S18†) by dynamic light scattering (DLS). The BDQ-NP showed a surface charge of +23.11 mV in water (Fig. S18†), suggesting interdigitation of cholesterol groups in the particle with positively charged ammonium groups on the surface. The BDQ-NP was stable with no change in size or PDI (Fig. S18†). The stability of the BDQ-NP was also demonstrated in PBS containing 10% fetal bovine serum over 48 hours (Fig. S19†).

Transmission electron microscopy (TEM) imaging supported the formation of BDQ-NP particles (Fig. 1b).^51^ In contrast to the self-assembly of amphiphilic BDQ into the BDQ-NP in aqueous solutions, hydrophobic BDS aggregated into larger and polydisperse BDS-NP in water (Fig. S16†). BDS-NP showed an average size of 460 nm (Fig. 1c) and a PDI of 0.27 by DLS. Singlet oxygen generation green (SOSG) assay showed both BDS-NP and BDQ-NP produced significant ^1^O_2_ signals under 660 nm LED irradiation in PBS solution with 0.5% (V/V) Triton-X-100 (Fig. 1d). This result shows the comparable ^1^O_2_ generation efficiency of BDS and BDQ molecules upon light irradiation.

### Subcellular location and MD simulation

**Fig. 2:**
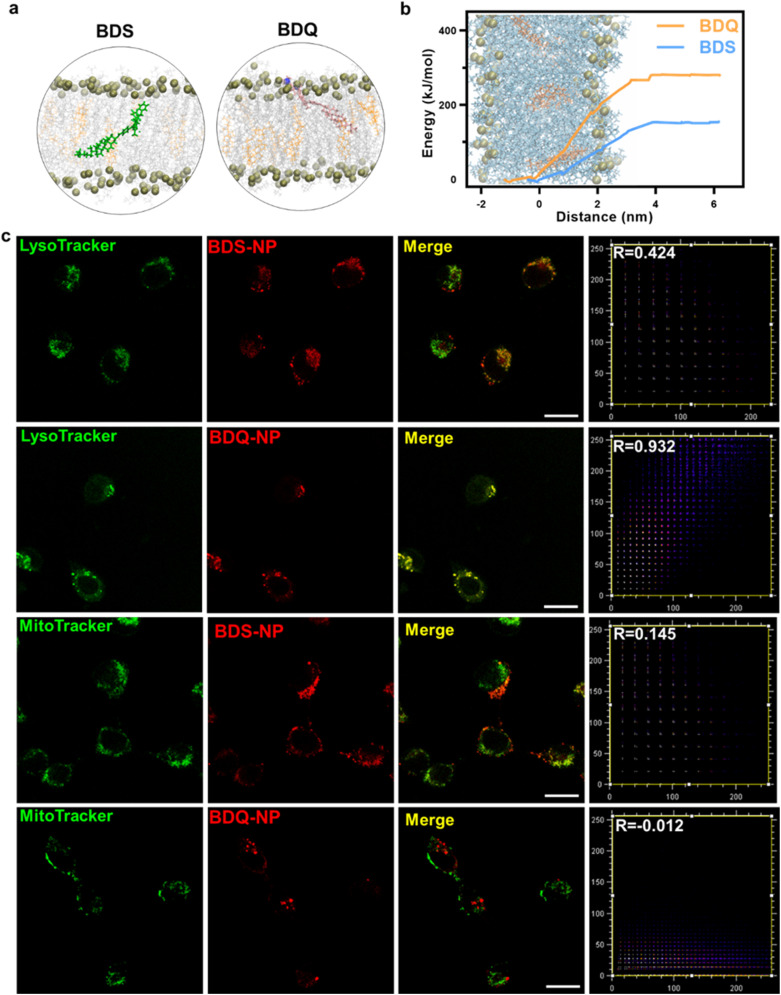
(a) MD simulation of BDS and BDQ in lipid bilayers. (b) Potentials of mean force of pulling BDS or BDQ molecules across the membrane to bulk water. (c) CLSM images showing colocalization between lysosomes or mitochondria and PSs after incubation of CT26 cells with the BDS-NP or BDQ-NP for 8 hours. Scale bar is 20 μm.

BDS-NP and BDQ-NP showed similar uptake in murine colon cancer CT26 cells (Fig. 1e). Subcellular localization of BDQ was studied by confocal microscopy. Most BDQ signals colocalized with LysoTracker (Pearson's coefficient *R* = 0.932) while only a small fraction of BDS signals colocalized with LysoTracker (*R* = 0.424) (Fig. 2c), suggesting lysosomal targeting by amphiphilic BDQ. Mitochondrial colocalization was also assayed with MitoTracker by confocal microscopy. BDQ showed almost no colocalization with MitoTracker (*R* = −0.012) while BDS showed low colocalization with MitoTracker (*R* = 0.145) (Fig. 2c). The different subcellular localization of BDS-NP and BDQ-NP likely result from their different uptake pathways: while the BDQ-NP maintains a stable micelle-like structure in aqueous solution and is taken up by cells *via* endocytosis, the BDS-NP is less stable in cell media and enters cells *via* both endocytosis and passive diffusion through the cell membranes. This finding was supported by MD simulation results (Fig. 2a and Movie S1†): cholesterol and cationic ammonium groups of BDQ strongly interact with hydrophobic interior and anionic phosphate head groups of the lysosome membrane, respectively (Movie S1†), which prevents BDQ leakage through the lipid bilayer. In contrast, MD simulation showed that BDS easily escaped through the lipid bilayer and translocated to other places (Fig. 2b, Movies S2 and 3†).

### Lysosome disruption, mitochondrial dysfunction, and cell death

**Fig. 3:**
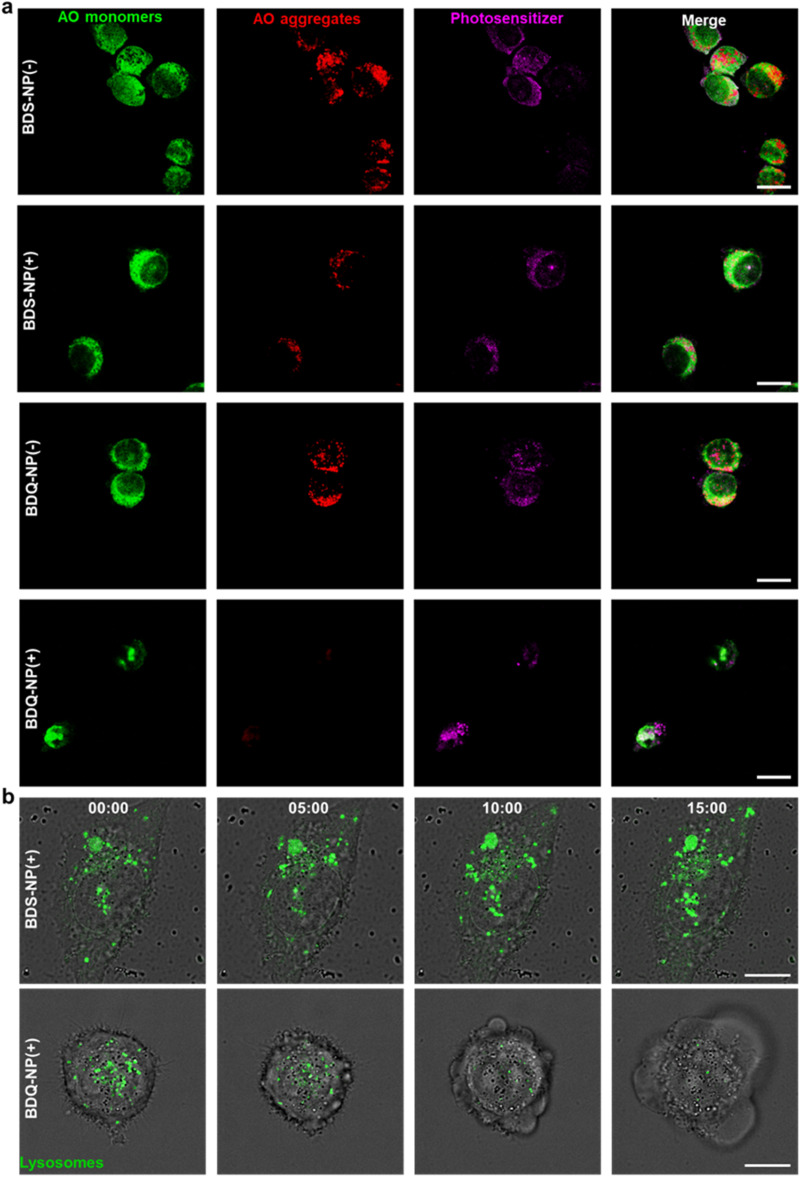
(a) CLSM images of lysosome targeting and disruption by AO staining in BDS-NP or BDQ-NP treated cells. (b) Snapshots of live confocal cell images at 0, 5, 10, and 15 minutes in BDS-NP or BDQ-NP treated CT26 cells. Scale bar is 20 μm in (a) and 10 μm in (b). (+) and (−) represent with or without irradiation, respectively.

The stable incorporation of BDQ into lysosome membranes resulted in severe lysosome disruption by BDQ-mediated PDT. Acridine orange (AO) labels acidic organelles by emitting red fluorescence from AO aggregates. At neutral pH, AO monomers show green fluorescence. With lysosome membrane disruption, lysosomes showed increased pH and reduced red fluorescence from AO. Confocal laser scanning microscopy (CLSM) imaging showed that the red fluorescence signals of BDQ-NP(+) treated cells completely disappeared while the red fluorescence in BDS-NP treated cells did not show significant change before or after light irradiation (Fig. 3a and S21†). BDQ-induced lysosome disruption was confirmed by flow cytometry (Fig. S22†). Without light irradiation, all groups showed high red fluorescence signals of acidic lysosomes. After light irradiation, PBS and BDS-NP-treated cells maintained high red signals, but BDQ-NP-treated cells showed steadily decreased lysosome signals that inversely correlated to BDQ-NP concentrations. Live cell imaging showed that light irradiation of BDQ-NP treated cells exhibited time-dependent lysosomal disruption and produced many bubbles at later time points, indicating the disintegration of these cells. In contrast, BDS-NP-treated cells showed unchanged lysosome signals and intact cells (Fig. 3b, Movies S4 and S5†).

**Fig. 4:**
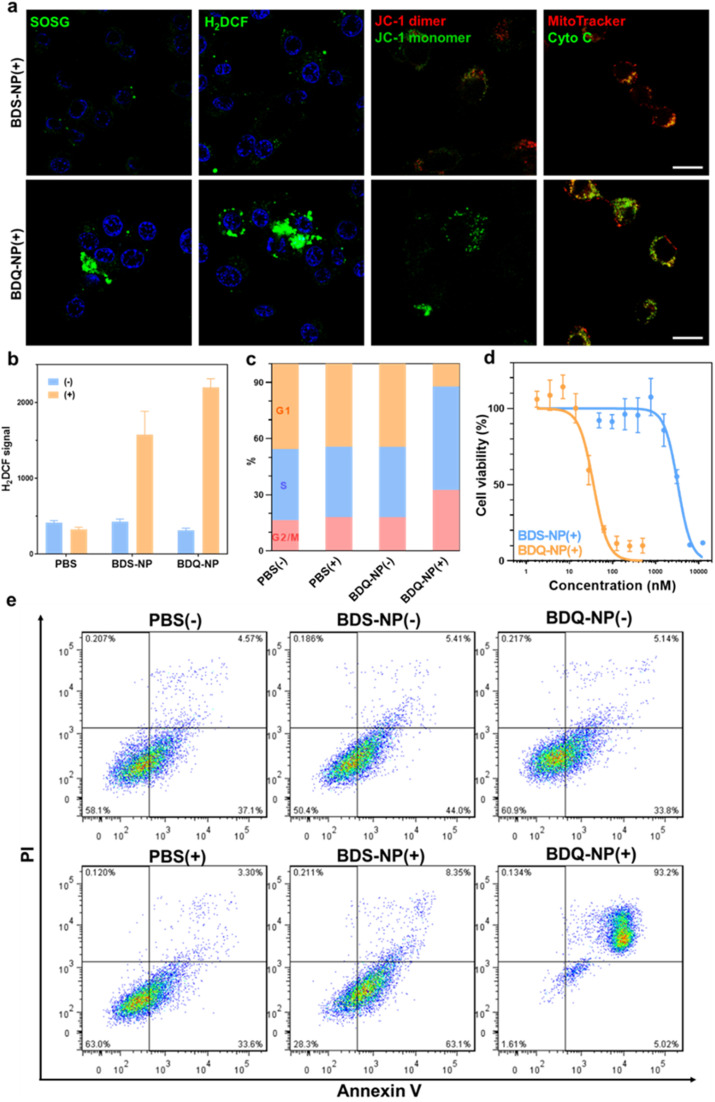
(a) Left to right, CLSM images of ^1^O_2_ by SOSG, total ROS generation by H_2_DCF, loss of mitochondrial membrane potential by JC-1, and release of cytochrome c of BDS-NP or BDQ-NP treated cells. Scale bar is 20 μm. (b) ROS generation by H_2_DCF assay by flow cytometry analysis. (c) Cell cycle study after different treatments. (d) Cytotoxicity by MTS assay. (e) Apoptosis/necrosis study by Annexin V/PI staining. (+) and (−) represent with or without irradiation (660 nm, 60 mW cm^−2^, 15 min).

Cells treated with the BDQ-NP(+) showed much higher ^1^O_2_ generation than those treated with the BDS-NP(+) by SOSG staining (Fig. 4a and S23†). The total ROS level in the cells was determined by H_2_DCF staining through CLSM imaging and flow cytometry (Fig. 4a, b and S24†). The BDQ-NP(+) showed a much higher level of ROS than BDS-NP(+). Mitochondrial dysfunction may trigger lysosomes and other organelles to further enhance ROS generation and promote cell death.^52^ CLSM studies showed depolarization of mitochondrial membrane potential and release of cytochrome c after BDQ-NP(+) treatment (Fig. 4a, S25 and S26†), indicating mitochondrial dysfunction. Cell cycle analysis showed that BDQ-NP(+) treated cells increased percentages of G2/M and S phases to 32.5% and 55.3%, respectively, from 16.4% and 38.0% for PBS(+) (Fig. 4c). Cell apoptosis/necrosis was confirmed by Annexin V/PI staining assay. 93.2% of BDQ-NP(+) treated cells were in the late apoptosis/necrosis stage while only 8.4% of BDS-NP(+) treated cells were in late apoptosis/necrosis (Fig. 4e). As a result, the BDQ-NP(+) showed an IC_50_ of 36.1 ± 4.8 nM, nearly 100 times lower than that of BDS-NP(+) (3.3 ± 0.3 μM) (Fig. 4d and S27†). Taken together, lysosome-targeting BDQ plus light irradiation causes lysosomal rupture and mitochondrial dysfunction to significantly increase cytotoxicity.

### *In vivo* biodistribution and antitumor effects

**Fig. 5:**
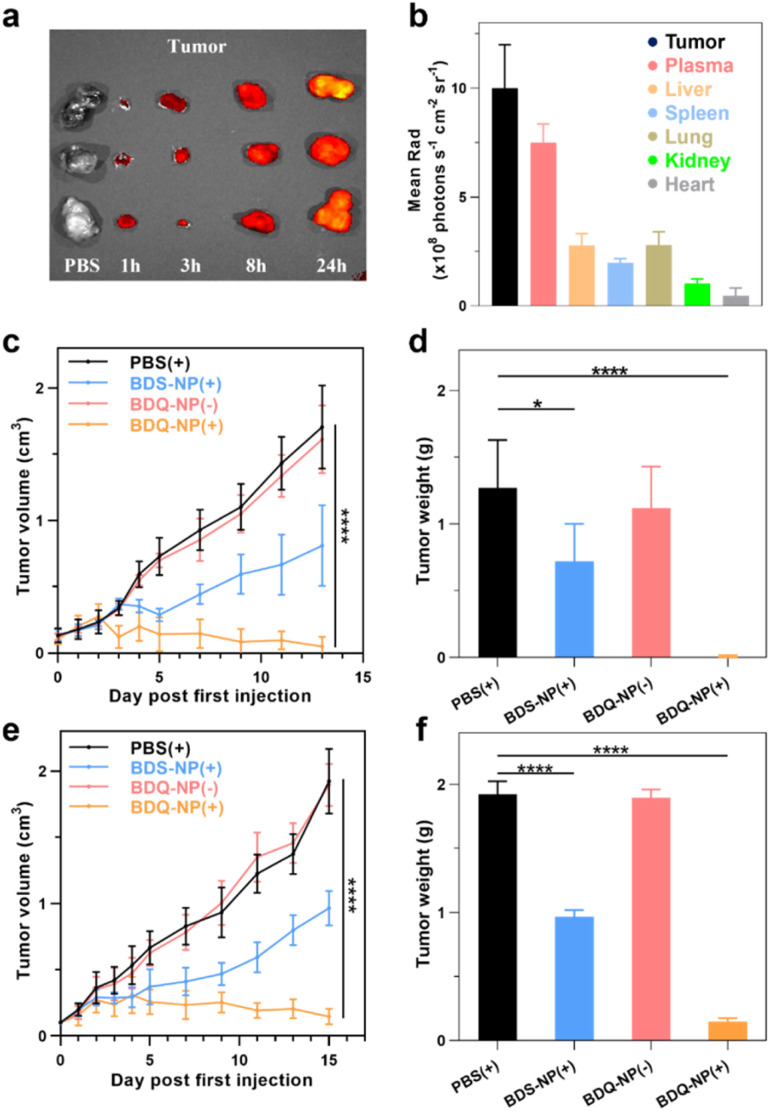
(a) BDQ fluorescence signals of CT26 tumors after PBS or BDQ-NP treatment by IVIS. (b) The mean fluorescence intensity of BDQ in different tissues of CT26 tumor-bearing mice at 24 hours post-injection. (c and e) Tumor growth curves of CT26 bearing mice (c) and orthotopic 4T1 bearing mice (e), *n* = 6. (d and f) Endpoint CT26 (d) or 4T1 (f) tumor weights after various treatments (*n* = 6). (+) and (−) represent with or without irradiation.

Biodistribution of the BDQ-NP was examined on CT26 tumor-bearing mice using an *in vivo* imaging system (IVIS) (Fig. S29†). The BDQ-NP did not show accumulation in hearts and exhibited low accumulation in kidneys. Lungs, spleens, and livers showed some BDQ-NP accumulation in the first 8 hours, but the signals significantly reduced at 24 h post-injection, suggesting systemic clearance of the BDQ-NP. In contrast, the signal of the BDQ-NP gradually increased in the tumors to reach a significantly higher level than other normal organs at 24 h post-injection (Fig. 5a and b). The BDQ-NP signal in the plasma remained high at 24 h post-injection, further supporting the long blood circulation of the BDQ-NP. The blood circulation half-life of the BDQ-NP was determined as 5.5 hours (Fig. S30†). The hemolysis test of the BDQ-NP did not cause appreciable hemolysis, with no hemolysis at 50 μM BDQ and <1% hemolysis at a very high BDQ concentration of 150 μM (Fig. S28†). Thus, the BDQ-NP shows a good passive targeting effect towards tumor tissues to minimize toxicity to normal tissues.

CT26 tumor-bearing mice were intravenously injected with 10 mg kg^−1^ BDQ-NP on days 0, 2, and 4 and tumors and irradiated (660 nm, 100 mW cm^−2^, 15 min) at 24 h post each injection. Tumor sizes were monitored and endpoint tumor weights were recorded (Fig. 5c and d). While 3 out of 6 BDQ-NP(+) treated mice were tumor-free and the other mice showed significant tumor regression (Fig. S31†), the BDQ-NP(−) did not show any efficacy. BDS-NP(+) treatment only moderately inhibited tumor growth. The tumor growth inhibition (TGI) indices were 99% and 43% for BDQ-NP(+) and BDS-NP(+) groups, respectively. All treatment groups showed steady body weights, suggesting minimal systemic toxicity from the treatments (Fig. S33a†).

### Lung metastasis inhibition

**Fig. 6:**
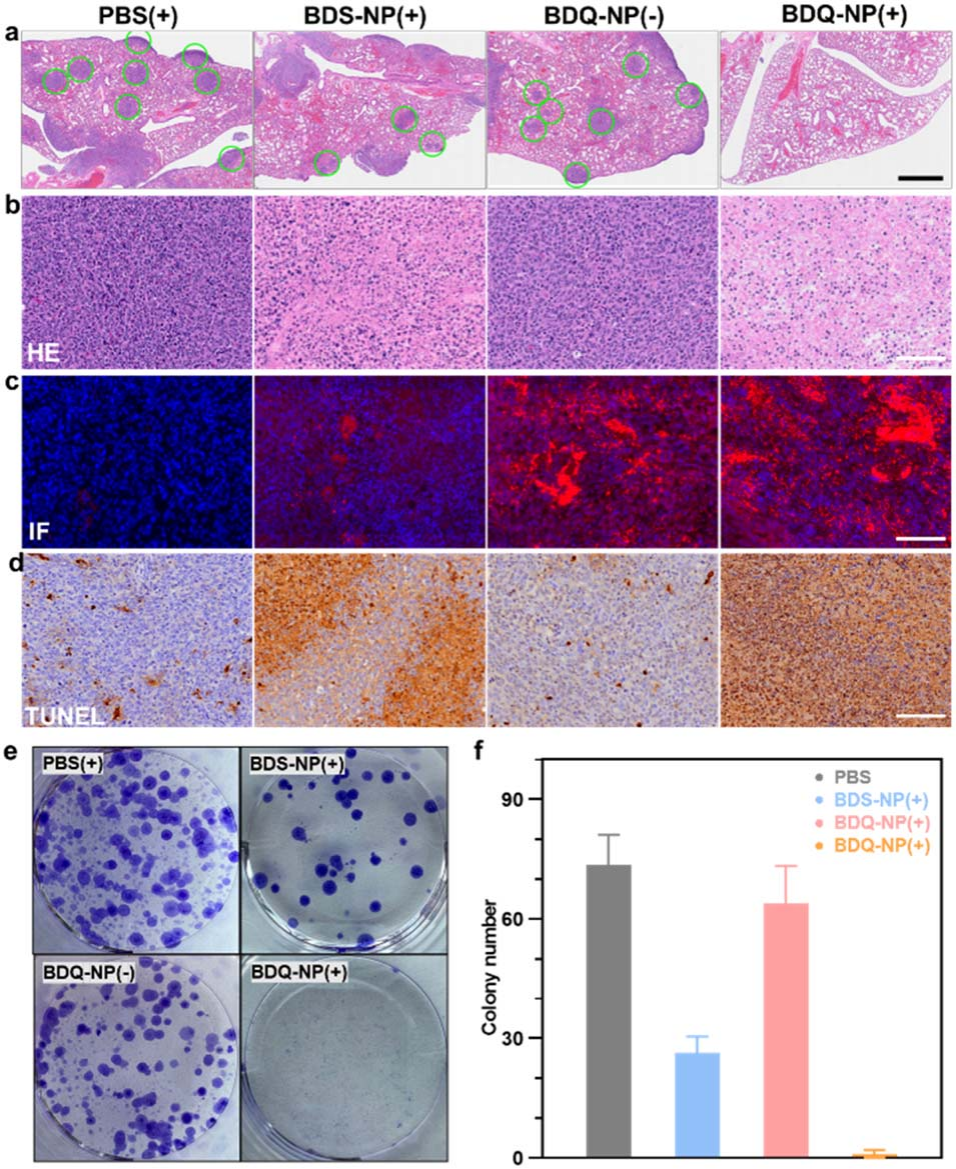
(a) Representative lung sections stained with H&E. Green circles indicate tumor nodules. Scale bar = 1 mm. (b–d) H&E staining (b), immunofluorescence (c) and TUNEL (d) of 4T1 tumor slices after different treatments. Scale bar is 100 μm. (e and f) Representative photos (e) and numbers (f) of colonies after digestion of the lungs from 4T1 tumor-bearing mice and culturing in the presence of 6-thioguanine for 12 days. (+) and (−) represent with or without irradiation.

Orthotopic 4T1 tumors in the mammary fat pads of mice produce spontaneous metastases to the lungs, making it a good breast cancer model to test antimetastasis effects. Orthotopic 4T1 tumor-bearing mice were intravenously injected with 10 mg kg^−1^ BDQ-NP on days 0, 2, and 4 and their tumors were irradiated at 24 h post each injection. The BDS-NP(+) slightly delayed 4T1 tumor progression with a TGI of 50% while the BDQ-NP(+) strongly inhibited 4T1 tumor growth with a TGI of 92% compared to the PBS control group (Fig. 5e, f and S32†). No weight loss was observed in all treated groups (Fig. S33b†). H&E staining of tumor sections showed severe necrosis in the BDQ-NP(+) group (Fig. 6b). The BDQ-NP infiltrated tumors much more than the BDS-NP (Fig. 6c). Immunohistochemistry showed high NLRP3, Caspase-1, and Caspase-3 expressions in BDQ-NP(+) treated tumors (Fig. S35†), indicating triggering of downstream events including inflammasome and caspase activation. Terminal deoxynucleotidyl transferase dUTP nick end labeling (TUNEL) assay showed strong apoptosis in resected tumors from the BDQ-NP(+) group (Fig. 6d). These results indicate that BDQ-NP(+) treatment induces massive cell death *via* inflammasome and caspase activation, showing the potential for further combination with systemic immunotherapies.

At the end of the study, tumor metastasis to the lungs was assessed. Compared to PBS control, the BDQ-NP(+) greatly suppressed lung metastasis (Fig. S34†). Lungs were digested and cultured in the presence of 60 μM 6-thioguanine for 12 days. After fixing with menthol, formed colonies were stained with 0.1% crystal violet. As 4T1 tumor cells are resistant to 6-thioguanine, only metastasized tumor cells in the lungs can proliferate and form colonies. The BDQ-NP(+) significantly reduced the colony number while PBS and BDQ-NP(−) groups formed numerous colonies (Fig. 6e and f). The lungs were further sectioned and stained by H&E (Fig. 6a). While PBS and BDQ-NP(−) groups had numerous tumor nodules, the BDQ-NP(+) group showed no tumor nodules. These results indicate that BDQ-NP(+) treatment efficiently prevents tumor metastasis to the lungs for practical cancer treatments.

## Conclusions

In this work, we designed a self-assembled BDQ-NP as a lysosome-targeting nano-photosensitizer. With stable incorporation of amphiphilic BDQ into the lipid bilayers of lysosomes, the BDQ-NP causes continuous LMP and generates a high level of ROS to disrupt mitochondrial functions and lead to high cytotoxicity at nanomolar concentrations under light irradiation. With good accumulation in tumours, the intravenously injected BDQ-NP showed excellent PDT efficacy on CT26 and orthotopic 4T1 tumour models without causing systemic toxicity. BDQ-NP-mediated PDT also efficiently prevented lung metastasis of 4T1 tumours. Our work highlights the potential of self-assembly of tumour-targeted nanoparticles from amphiphilic photosensitizers for organelle-specific PDT.

## Data availability

All data are available in the ESI.†

## Author contributions

Youyou Li: conceptualization, methodology, investigation, writing — original draft. Wenbo Han: conceptualization, methodology, investigation, writing — original draft. Deyan Gong: investigation, data curation. Taokun Luo: investigation. Yingjie Fan: investigation. Jianming Mao: software. Wenwu Qin: writing — review & editing. Wenbin Lin: resources, writing — review & editing, supervision, funding acquisition.

## Conflicts of interest

The authors declare no competing interest.

## Supplementary Material

SC-014-D3SC00455D-s001

SC-014-D3SC00455D-s002

SC-014-D3SC00455D-s003

SC-014-D3SC00455D-s004

SC-014-D3SC00455D-s005

SC-014-D3SC00455D-s006
