# Supplementary material for: A self-assembled nanophotosensitizer targets lysosomes and induces lysosomal membrane permeabilization to enhance photodynamic therapy
Source: Chem Sci. 2023 Apr 19;14(19):5106–15. doi: 10.1039/d3sc00455d (PMC10189857; doi:10.1039/d3sc00455d)
Supplement: SC-014-D3SC00455D-s001 [file SC-014-D3SC00455D-s001.pdf]

Electronic Supplementary Material (ESI) for Chemical Science.
This journal is © The Royal Society of Chemistry 2023

Supporting Information for

Self-Assembled Nanophotosensitizer Targets Lysosomes and Induces Lysosomal Membrane Permeabilization to Enhance Photodynamic Therapy

Youyou Li[†a], Wenbo Han[†a], Deyan Gong[c], Taokun Luo[a], Yingjie Fan[a], Jianming Mao[a], Wenwu Qin[c], and Wenbin Lin*[ab]

[a] Department of Chemistry, The University of Chicago, Chicago, Illinois 60637, United States. E-mail: wenbinlin@uchicago.edu

[b] Department of Radiation and Cellular Oncology and Ludwig Center for Metastasis Research, The University of Chicago, Chicago, IL 60637, United States

[c] Key Laboratory of Nonferrous Metal Chemistry and Resources Utilization of Gansu Province and State Key Laboratory of Applied Organic Chemistry, College of Chemistry and Chemical Engineering, Lanzhou University, Lanzhou 730000, China.

† These authors contributed equally to this work.

Materials and Instruments. All starting materials were purchased from Sigma-Aldrich and Fisher (USA) unless otherwise noted, and used without further purification. ^{1}H NMR spectra were recorded on a Bruker NMR 500 DRX spectrometer at 500 MHz or Bruker Avance III HD nanobay 400 MHz at 400MHz as labeled and referenced to the proton resonance resulting from incomplete deuteration of $CDCl_3$ or DMSO-d_6. Matrix-assisted laser desorption/ionization-time of flight high-resolution mass spectrometry (MALDI-TOF HRMS) data were collected on a Bruker Ultraflextreme MALDI-TOF/TOF using positive ion mode. Transmission electron microscopy (TEM) was carried out on a TECNAI Spirit instrument. Ultraviolet-visible absorption spectra (UV-Vis) were acquired with a Shimadzu UV-2600 UV-Vis spectrophotometer. Luminescence data were measured using an RF-5301PC spectrofluorophotometer (Shimadzu, Japan) and a Xenogen IVIS 200 imaging system (Xenogen, USA). Particle sizes and zeta potentials of nano-photosensitizers were measured via dynamic light scattering (DLS) and electrophoresis with a Malvern Nano Series Zeta-Sizer. Flow cytometry data were collected on an LSR-Fortessa 4-15 (BD Biosciences) and analyzed by FlowJo software (Tree Star, Ashland, OR). Confocal laser scanning microscope images were collected on Leica SP8 and analyzed with ImageJ software. IVIS imaging was performed using the IVIS Kinetic Imaging System (Caliper Life Sciences, Hopkiton, MA). All imaging parameters were kept constant for the whole imaging study.

Cell Lines and Animals. Murine colon adenocarcinoma CT26 cells and murine metastatic triple-negative breast carcinoma 4T1 were purchased from the American Type Culture Collection (Rockville, MD, USA) and cultured in RPMI-1640 (Corning, USA) supplemented with 10% fetal bovine serum, 100 U/mL penicillin G sodium and 100 μg/mL streptomycin sulfate in a humidified atmosphere containing 5% CO2 at 37 °C. BALB/c female mice (6 weeks, 18-22 g) were provided by Harlan Laboratories, Inc (USA). The study protocol was reviewed and approved by the Institutional Animal Care and Use Committee (IACUC) at the University of Chicago. The Human Tissue Resource Center at the University of Chicago provided histology-related services for this study.

Statistical analysis. All statistical analyses were performed by the One-way Repeated Measures ANOVA method with Tukey's honest significance test to determine whether the difference between each group was significant. The p values were defined as * $p<0.05$, ** $p<0.01$, *** $p<0.001$ in all figures.

Synthesis of BOD. BOD was synthesized by the previously reported method.[1] Briefly, 4-carboxybenzaldehyde aldehyde (525.5 mg, 3.5 mmol) and 400 mL anhydrous dichloromethane

were added to a 500 mL round bottom flask. The mixture was degassed by nitrogen for 30 min and then 2,4-dimethyl pyrrole (721 μl, 7 mmol) was added. 100 μl TFA was added and the solution was stirred under N_2 overnight at dark at room temperature. After the addition of a solution of DDQ (794.5 mg, 3.5 mmol) in 100 mL dichloromethane to the reaction mixture, stirring was continued for 4 hours. Then 10 mL Et_3N and 10 mL $BF_3 \cdot OEt_2$ were successively added in the ice bath. After 12 hours, the reaction mixture was treated with 300 mL water and the organic layer was dried over anhydrous Na_2SO_4. The solvent was evaporated and the residue was purified by silica gel column chromatography (avoid light). Orange solid was obtained. ^{1}H NMR (400 MHz, DMSO) δ 13.22 (s, 1H), 8.09 (d, J = 10.3 Hz, 2H), 7.51 (d, J = 10.2 Hz, 2H), 6.18 (s, 2H), 2.44 (s, 6H), 1.33 (s, 6H). HRMS: m/z=367.148([M]$^-$).

Synthesis of I_2-BOD. To a solution of BOD (543 mg, 1.48 mmol) and iodine (980 mg, 3.70 mmol) in 10 mL EtOH was added 5.5 mL iodic acid (543 mg, 2.96 mmol) in an aqueous solution. The mixture was stirred at 60 °C for 1 h. After cooling to room temperature, saturated $Na_2S_2O_3$ solution was added and the product was extracted with CH_2Cl_2. The CH_2Cl_2 solution was evaporated and the residue was purified by silica gel column chromatography to afford I_2-BOD as a red solid (916 mg, 100% yield). ^{1}H NMR (500 MHz, $CDCl_3$) δ 8.29 (d, J = 8.5 Hz, 2H), 7.44 (d, J = 8.2 Hz, 2H), 2.66 (s, 6H), 1.39 (s, 6H). HRMS: m/z=618. 938([M]$^-$).

Synthesis of Chol-I_2-BOD. Carbonyldiimidazole (CDI) (71 mg, 0.44 mmol) was added to a solution of I_2-BOD (180 mg, 0.29 mmol) in 10 mL of dry THF under continuous stirring at room temperature. The mixture was stirred at room temperature for 1 h. To this mixture under continuous stirring was then added a solution of cholesterol (Chol, 170 mg, 0.44 mmol) and NaH (40 mg, 1.67 mmol) in 10 mL dry THF over 20 min at room temperature through a 0.2 μm polytetrafluoroethylene (PTFE) syringe driven filter. The mixture solution was heated at 80 °C overnight. After the removal of solvents, the dry pink solid was purified by silica gel column chromatography [hexanes/ethyl acetate, 10:1 (v/v)] to afford Chol-I_2-BOD as a red solid (229 mg, 80% yield). ^{1}H NMR (500 MHz, $CDCl_3$) δ 8.20 (d, J = 8.4 Hz, 2H), 7.36 (d, J = 8.4 Hz, 2H), 5.45 (d, J = 3.4 Hz, 1H), 4.92 (m, 1H), 2.65 (s, 6H), 1.37 (s, 6H), 2.60 – 0.50 (43H). ^{13}C NMR (101 MHz, $CDCl_3$) δ 169.58, 144.05, 140.91, 140.67, 138.30, 121.87, 119.87, 98.50, 90.75, 88.47, 77.48, 77.36, 77.16, 76.84, 74.05, 71.96, 67.89, 59.62, 59.39, 58.08, 56.92, 56.30, 50.28, 42.47, 42.45, 39.93, 39.66, 37.40, 36.65, 36.33, 35.93, 33.85, 32.06, 29.84, 28.38, 28.16, 26.16, 24.94, 24.44, 23.97, 22.97, 22.71, 21.23, 19.54, 18.86, 15.33, 12.01, 1.16. HRMS: m/z=988.295 ([M]$^+$).

Synthesis of Chol-I_2-styryl-BOD (BDS)

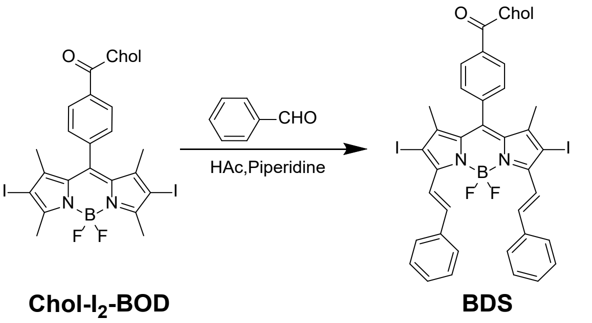

To a solution of Chol-I_2-BOD (140 mg, 0.14 mmol) and benzaldehyde (60 μL, 0.56 mmol) in 10 mL dry toluene in a thick-walled pressure bottle with molecular sieve, 710 μL glacial acetic acid and 710 μL piperidine were added. The mixture was heated in the closed bottle at 115 °C for 2.5 h. After cooling to room temperature, organic volatiles was evaporated. The residue was purified by silica gel column chromatography [hexanes/ethyl acetate, 10:1 (v/v)]. The resultant solid was dissolved in a small amount of THF and recrystallized from diethyl ether to afford pure BDS as a dark blue solid (81 mg, 50% yield). ^{1}H NMR (500 MHz, $CDCl_3$) δ 8.23 (d, J = 8.6 Hz, 2H), 8.17 (d, J = 16.7 Hz, 2H), 7.73 – 7.66 (m, 6H), 7.45 – 7.40 (m, 6H), 7.36 (t, J = 7.3 Hz, 2H), 5.46 (d, J = 3.5 Hz, 1H), 4.97 – 4.88 (m, 1H), 1.45 (s, 6H), 2.60 – 0.50 (43H). ^{13}C NMR (101 MHz, $CDCl_3$) δ 150.18, 140.17, 136.72, 136.40, 135.92, 130.79, 129.87, 129.55, 128.99, 128.75, 127.95, 126.78, 126.50, 125.68, 123.20, 118.86, 56.86, 56.31, 50.22, 42.50, 39.90, 39.68, 38.38, 37.20, 36.84, 36.35, 35.96, 34.47, 34.38, 32.91, 32.25, 32.08, 30.47, 30.32, 30.19, 30.12, 29.86, 29.82, 29.75, 29.52, 29.23, 28.40, 28.18, 28.06, 27.24, 26.86, 24.62, 24.46, 23.99, 23.33, 22.98, 22.85, 22.72, 21.23, 21.01, 19.89, 19.55, 18.89, 17.92, 14.27, 12.03. HRMS: m/z=1164.350 ($[M]^+$).

Synthesis of BDS-(N^+Me_3)$_2$ (BDQ). To a solution of Chol-I_2-BOD (140 mg, 0.14 mmol) and 4-dimethylaminobenzaldehyde (350 mg, 2.35 mmol) in 10 mL dry toluene in a thick-walled pressure bottle with molecular sieve, 710 μL glacial acetic acid and 710 μL piperidine were added. The mixture was heated in the closed bottle at 115 °C for 4 h. After the removal of organic volatiles, the residue was purified by silica gel column chromatography with [$CHCl_3$/MeOH =15:1 (v/v)]. The solid product was recrystallized in diethyl ether to afford the BDS-(NMe_2)$_2$ intermediate as a gray solid (105 mg, 60% yield), which was directly used in the subsequent step. A solution of BDS-(NMe_2)$_2$ (100 mg, 0.08 mmol) and methyl iodide (2 mL, 32 mmol) in 10 mL dry THF in a closed thick-walled pressure bottle was heated at 70 °C for 2 days. The solvents were evaporated. The solid was suspended in CH_2Cl_2 and then centrifugated at 14,000 rpm for 15 min. The solid was dissolved in DMF and then centrifugated at 14,000 rpm for 15 min. The solution was evaporated to afford BDS-(N^+Me_3)$_2$(BDQ) as a dark blue

solid (110 mg, 0.07 mmol, 90% yield). ^{1}H NMR (500 MHz, DMSO) δ 8.19 (d, *J* = 8.4 Hz, 2H), 8.08 – 8.03 (m, 6H), 7.89 (d, *J* = 9.3 Hz, 4H), 7.68 (d, *J* = 8.4 Hz, 2H), 7.57 (d, *J* = 16.8 Hz, 2H), 5.43 (s, 1H), 4.82 – 4.74 (m, 1H), 3.64 (s, 18H), 1.42 (s, 6H), 2.60 – 0.50 (43H). ^{13}C NMR (101 MHz, DMSO) δ 150.33, 147.44, 146.36, 139.38, 137.39, 137.20, 132.58, 130.36, 128.88, 128.47, 128.19, 124.89, 122.42, 121.48, 120.74, 111.71, 74.74, 56.43, 56.11, 55.59, 49.44, 41.86, 37.98, 36.53, 36.15, 35.63, 35.20, 34.36, 31.39, 30.40, 29.76, 29.32, 29.02, 27.77, 27.39, 23.89, 23.21, 22.66, 22.39, 21.02, 20.57, 19.00, 18.54, 17.38, 11.68. HRMS: m/z=640.2514 ($[M]^{2+}$).

Critical micellar concentration (CMC) determination. The fluorescence intensities of BDQ aqueous solutions with different concentrations were recorded using a fluorophotometer (Shimadzu) at an excitation wavelength of 355 nm and an emission wavelength of 660 nm. The CMC was estimated as the extrapolated cross-point of the fitting curves for fluorescence intensities to concentrations.

Cellular uptake. 10^5 CT26 cells were seeded in a 6-well plate and incubated with 100 nM BDS-NP or BDQ-NP for 8 h. The cells were collected and washed with PBS three times before analyzed by flow cytometry.

Subcellular localization. 10^5 CT26 cells were seeded in dishes and incubated with 40 nM BDS-NP or BDQ-NP for 8 h. After being washed with PBS three times, the cells were stained with 100 nM LysoTracker™ Green DND-26 or MitoTracker™ Red CMXRos (Thermo Fisher Scientific, USA) for 15 min and then washed with PBS three times before imaging under CLSM. Colocalization was evaluated by Pearson's coefficient.[2]

Molecular Dynamics Simulations.

Molecular dynamic simulations were carried out according to reported procedures[3] using Gromacs 2021.1 package.[4] The DMPC bilayer, which was pre-equilibrated for 400 ns, was taken from the SLipids force field.[5] The system composition is shown in Table S1. The system was simulated with periodic boundary conditions as in the simulation setup of the lipid bilayer. The Slipid force field was selected as it is considered one of the best all-atom force fields for lipid systems. The structures of BDS and BDQ were optimized by Gaussian16 at the B3LYP/6-31++G(d) level of theory and RESP partial charges were added to the topologies. Acpype[6] was used to generate the input topologies for Gromacs.

All simulations were performed in NPT conditions with the constant pressure of 1 bar and the temperature of 303 K maintained by v-rescale thermostat and Berendsen barostat,

respectively. First, the BDS or BDQ molecule was positioned in the bulk water and equilibrated for 1 ns (Figure S12). For the production run, long-range electrostatic interactions were treated using the particle-mesh Ewald scheme[7] with a grid spacing of 1.6 Å and a cut-off 12 Å was applied for the Van der Waals interactions. All hydrogen related bonds were constrained using the LINCS algorithm. The systems were then equilibrated for 70 ns and 250 ns with a time step of 2 fs for BDS and BDQ, respectively, to acquire stable configurations of insertion into DMPC membrane.

Potentials of mean force (PMFs) of pulling BDS and BDQ across the membrane were computed by umbrella sampling simulations. The center masses of BDS and BDQ were restrained by harmonic potential characterized by a 1000 kJ mol^{-1} nm^{-2} force constant at different distances from the center of the slice. Seven sampling windows were selected with a step interval of 1 nm along the Z axis. Each window was sampled for 50 ns and the last 10 ns were used for statistical analysis. PMFs were obtained by weighted histogram technique[8] in Gromacs package. All trajectories were visualized by VMD.[9]

Lysosome disruption by AO staining. 10^5 CT26 cells were seeded in dishes and incubated with 40 nM BDS-NP or BDQ-NP for 8 h, then irradiated (660 nm, 60 mW/cm^2, 15 min), washed with PBS three times and stained with 1 μM acridine orange (AO) for 15 min. The cells were washed with PBS three times to remove excess probes and immediately observed by CLSM. For flow cytometry experiments, the cells were seeded in 6-well plates ($5x10^5$ cells per well) and treated with BDS-NP or BDQ-NP at different concentrations for 8 h. The control experiments without light irradiation were similarly performed.

Lysosome disruption by live imaging. 10^5 CT26 cells were seeded in glass bottom dishes and incubated with 40 nM BDS-NP or BDQ-NP for 8 h. The cells were washed with PBS for three times and stained with 500 nM LysoTracker™ Green DND-26 (Thermo Fisher Scientific, USA) in serum-free RMPI-1640 medium for 45 min at 37 °C. The cells were then rewashed with PBS and exchanged into warm complete RPMI-1640 medium for 15 min at 37 °C. Afterward, the cells were washed with PBS and exchanged into phenol red-free RPMI-1640 medium for live imaging. The laser at 660 nm from Leica Stellaris 8 confocal microscope was used as an *in situ* excitation source for BDQ-NP or BDS-NP. The laser power was measured by a photometer and normalized to 60 mW/cm^2. The cells were monitored in the bright field and lysotracker channel with continuous 660 nm laser irradiation at room temperature.

Singlet oxygen and ROS generation. 10^5 CT26 cells were seeded in dishes and incubated with 40 nM BDS-NP or BDQ-NP for 8 h, washed with PBS three times and stained with Singlet

Oxygen Sensor Green (SOSG) for 15 min, then irradiated (660 nm, 60 mW/cm^2, 15 min). The control groups were not irradiated. The cells were washed with PBS three times to remove excess probes and immediately observed by CLSM. For ROS generation, the cells were stained with the H_2DCFDA assay.

Mitochondrial membrane potential change. 10^5 CT26 cells were seeded in dishes and incubated with 40 nM BDS-NP or BDQ-NP for 8 h, then irradiated (660 nm, 60 mW/cm^2, 15 min). After being washed with PBS three times and stained with 10 mM JC-1 for 15 min, the cells were washed with PBS three times to remove excess probes and immediately observed by CLSM.

Release of cytochrome c. 10^5 CT26 cells were seeded in dishes and incubated with 40 nM BDS-NP or BDQ-NP for 8 h, then irradiated (660 nm, 60 mW/cm^2, 15 min). After being washed with PBS three times, the cells were stained with 100 nM MitoTracker Red CMXROS for 15 min. The cells were then washed with PBS three times and fixed with 3.7% paraformaldehyde (PFA) for 15 min. The cells were washed with PBS three times, blocked by 2% FBS for 15 min and permeabilized by 0.2% Triton-X for 15 min. Finally, the cells were incubated with FITC-conjugated cytochrome c antibody (Thermo Fisher Scientific, USA) 1:100 in 2% BSA PBS solution at 4°C for 2 hours. The cells were washed with PBS three times and observed under CLSM immediately.

Cell cycle analysis. 10^5 CT26 cells were seeded in 6 well plates and incubated with 40 nM BDS-NP or BDQ-NP for 8 h, then irradiated (660 nm, 60 mW/cm^2, 15 min). The cells were further incubated for 24 h and harvested. Harvested cells were washed with PBS three times and fixed with 70% ethanol for 1 h at 4 °C. Ethanol was then removed, and fixed cells were stained with staining buffer for 15 min at room temperature in dark before cell cycle analysis using flow cytometry.

Cytotoxicity. CT26 cells were seeded in 96-well plate (2x10^3 cells/well) and incubated with different concentrations of BDS-NP or BDQ-NP for 8 h, then irradiated (660 nm, 60 mW/cm^2, 15min). The cells were further incubated for another 48 h and cell viability was tested by MTS assay.

Apoptosis and necrosis. 10^5 CT26 cells were seeded in 6 well plates and incubated with 40 nM BDS-NP or BDQ-NP for 8 h, then irradiated (660 nm, 60 mW/cm^2, 15 min). The cells were further incubated in fresh medium for 24 h. The cells were harvested and washed with PBS three times, then stained with Annexin V and PI for 15 min before analysis by flow cytometry.

Hemolysis assay. Fresh mouse blood was thoroughly washed with PBS to obtain erythrocytes. Then 5% erythrocytes in PBS (negative group), PBS solutions containing different concentrations of BDQ-NP, and 0.1% Trion-X-100 water solution (positive group) were incubated at 37 °C for 1 hour. Then samples were centrifuged, and the absorbances for the supernatants were recorded at 540 nm (OD). Hemolysis rates were calculated as $(OD_{sample} - OD_{negative})/(OD_{positive}-OD_{negative}) \times 100\%$.

Biodistribution. 10 mg/kg BDQ-NP was intravenously dosed to 8-week-old CT26 tumor-bearing mice. Mice were sacrificed at different time points. Plasma, tumors, and other organs were collected and imaged by IVIS using the Cy5.5 channel.

***In vivo* efficacy on CT26 tumor-bearing mice.** CT26 cells (2×10^6) were injected subcutaneously into BALB/c mice. When tumors reached around 100 mm^3 on day 7 post-tumor inoculation, 10 mg/kg BDS-NP or BDQ-NP was intravenously injected once every 2 days for 3 doses. Twenty-four hours post each drug administration, mice were anesthetized with isoflurane, and the tumors were irradiated (660 nm, 100 mW/cm^2, 15 min) for irradiation groups. Tumor volumes were calculated by (width × length × width)/ 2. Body weights of mice were monitored. Mice were sacrificed 13 days after the first injection when the tumors of the control group reached 2 cm^3. Excised tumors were collected, photographed, and weighed. TGI was calculated as (1 – (mean volume of treated tumors)/(mean volume of control tumors)) × 100%.

***In vivo* efficacy on orthotopic 4T1 tumor-bearing mice.** 4T1 cells (2×10^5) were injected into female BALB/c mice mammary pads. When tumors reached around 100 mm^3 on day 7 post-tumor inoculation, 10 mg/kg BDS-NP or BDQ-NP was *i.v.* injected once every 2 days for 3 doses. Twenty-four hours post each drug administration, mice were anesthetized with isoflurane, and the tumors were irradiated (660 nm, 100 mW/cm^2, 15 min) for irradiation groups. Tumor volumes were calculated by (width × length × width)/2. Body weights of mice were monitored. Mice were sacrificed 15 days after the first injection when the tumors of the control group reached 2 cm^3. Excised tumors were photographed, weighed and sectioned for hematoxylin-eosin (H&E) staining, and immunofluorescence and immunohistochemistry analysis. Metastatic tumors were found in lungs when dissecting mice. Lungs were also harvested, photographed for tumor nodules, then sectioned and stained with H&E, or digested with collagenase type IV/elastase cocktail and cultured with 60 μM 6-thioguanine for 12 days.[10] The colonies formed by clonogenic metastatic tumor cells were then fixed with methanol and stained with 0.1% crystal violet. Number of colonies was counted.

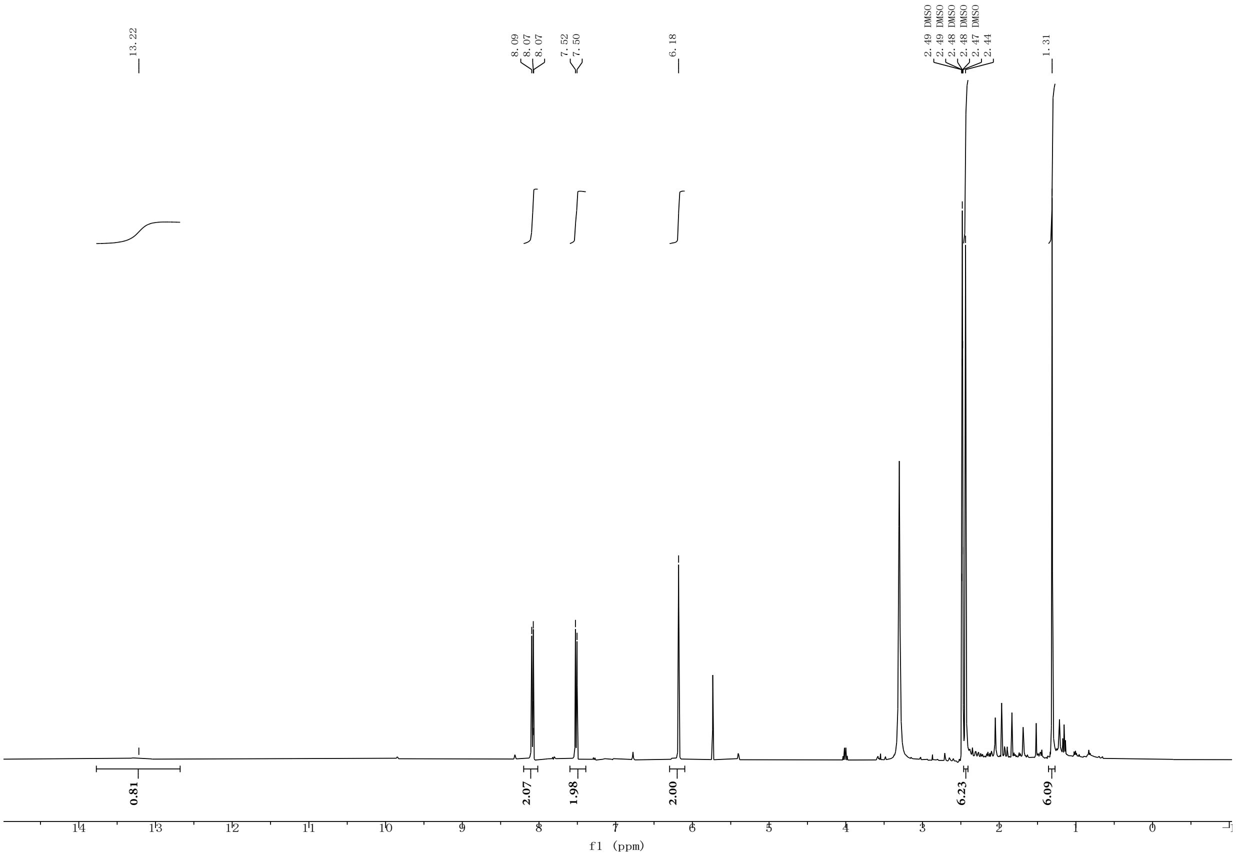

Figure S1. ^{1}H NMR spectrum of BOD (DMSO, 400 MHz).

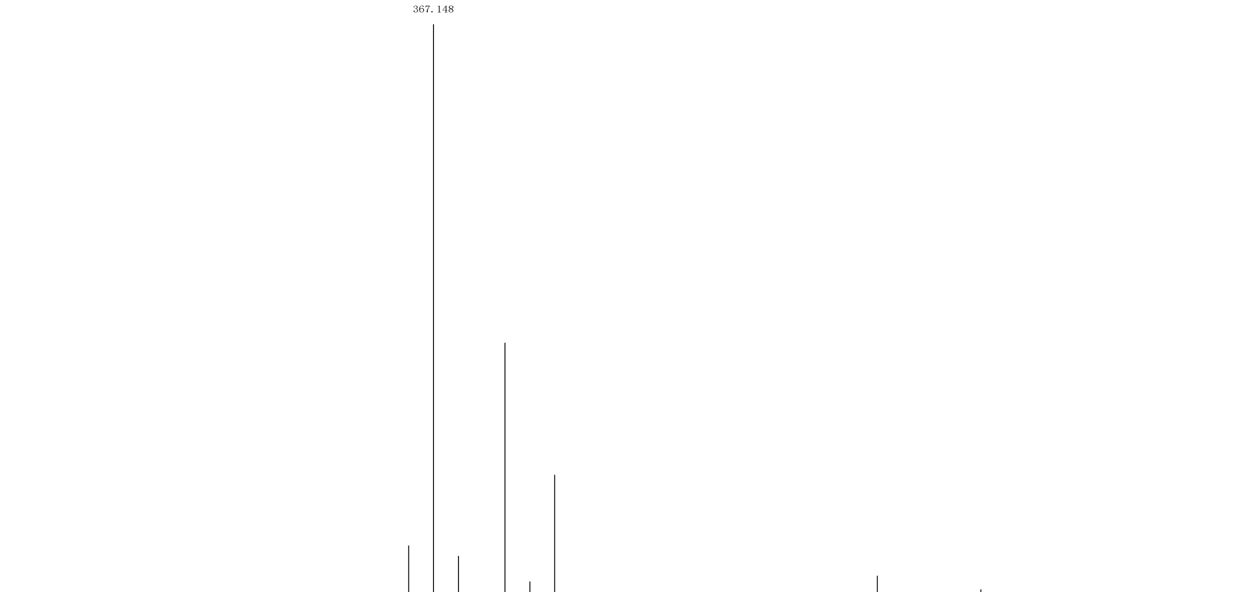

Figure S2. Mass spectrum of BOD.

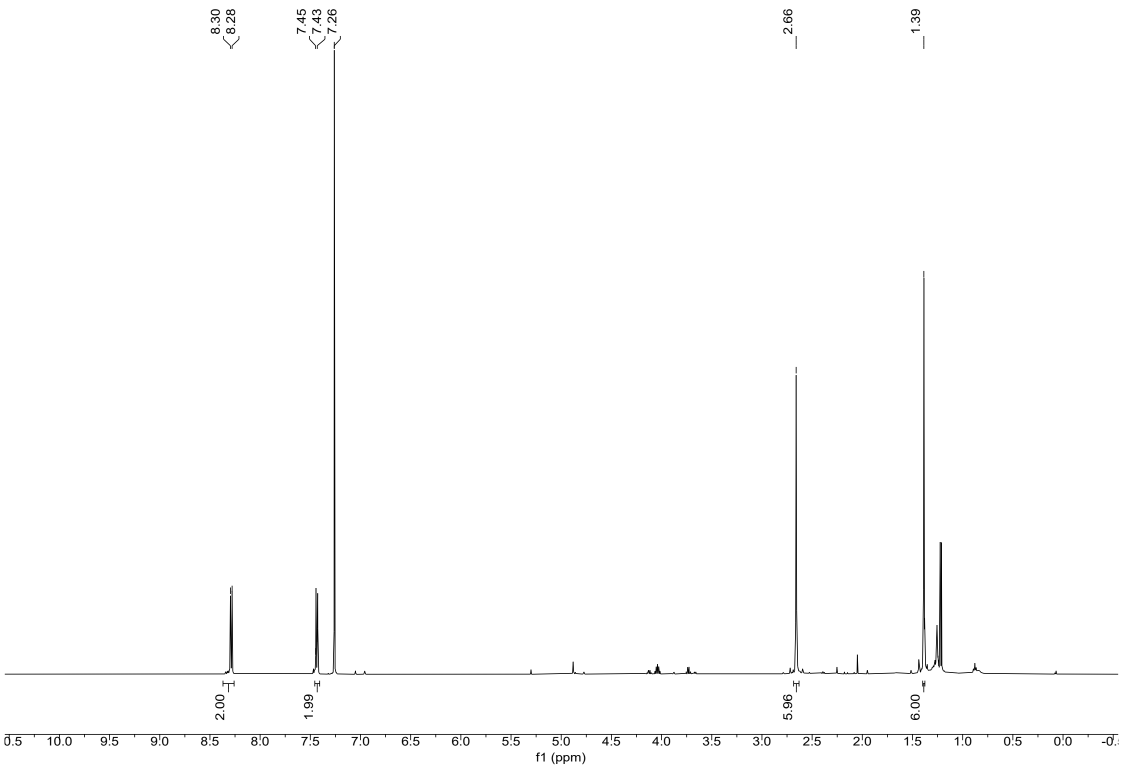

Figure S3. ^{1}H NMR spectrum of I_2-BOD ($CDCl_3$, 500 MHz).

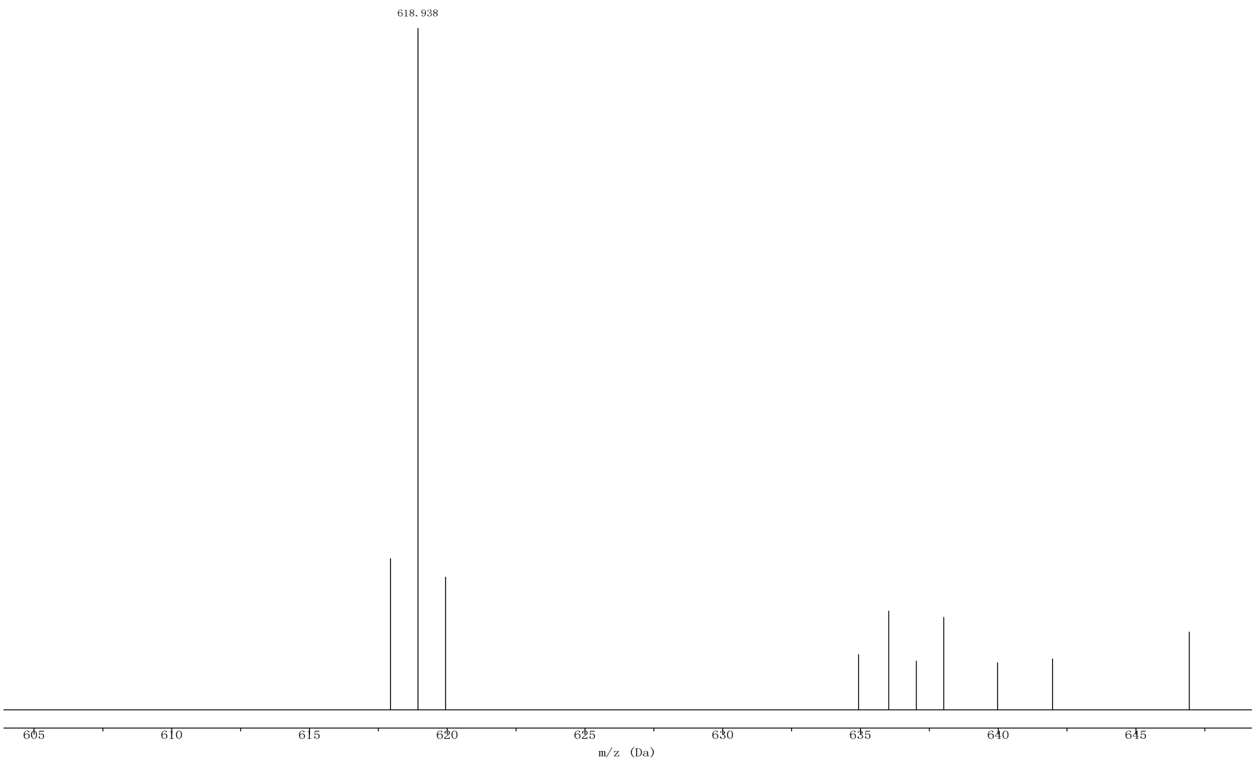

Figure S4. Mass spectrum of I_2-BOD.

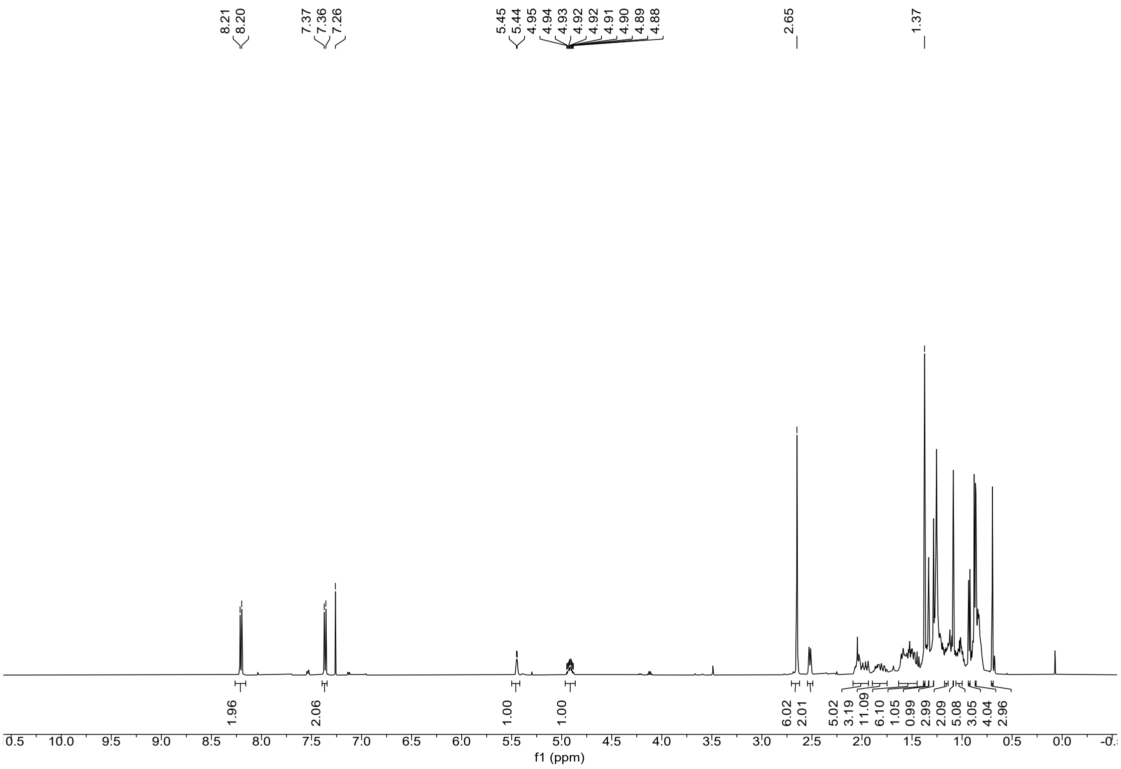

Figure S5. ^{1}H NMR spectrum of Chol-I_2-BOD ($CDCl_3$, 500 MHz).

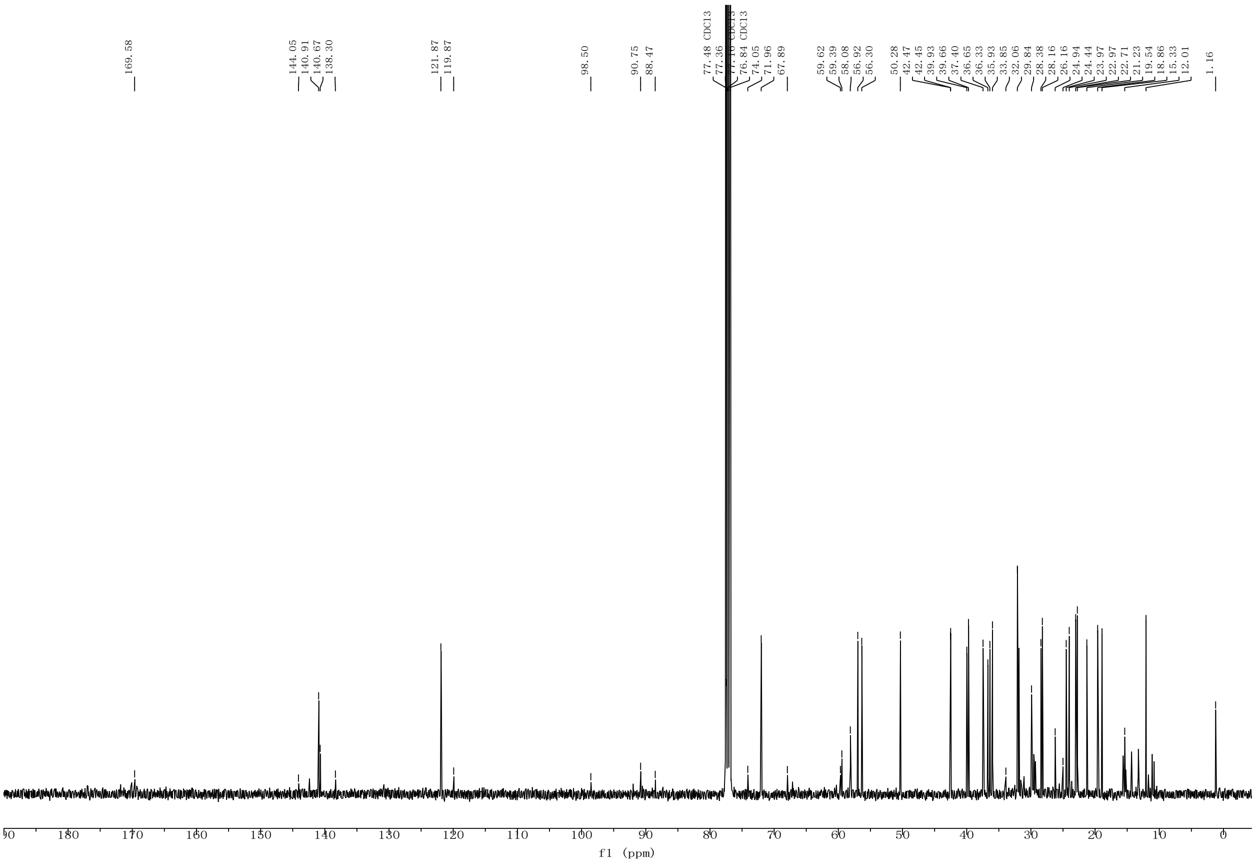

Figure S6. ^{13}C NMR spectrum of Chol-I_2-BOD ($CDCl_3$, 101 MHz).

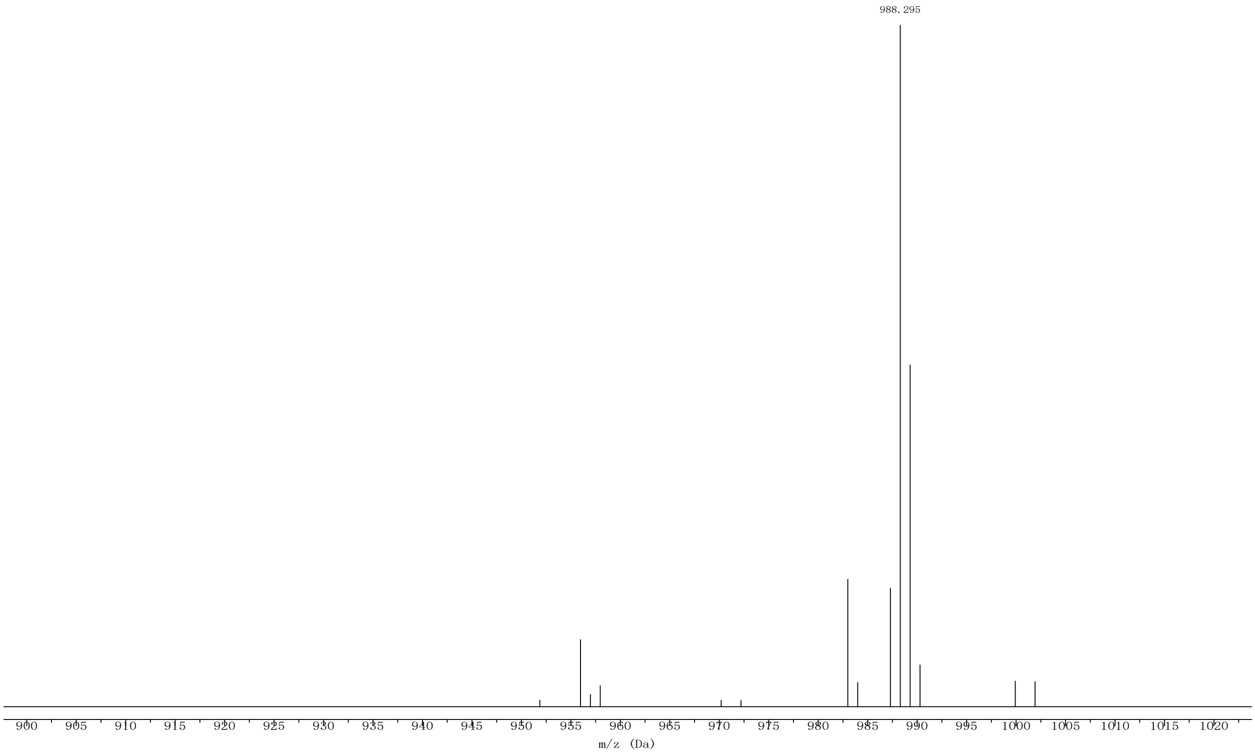

Figure S7. Mass spectrum of Chol-I_2-BOD.

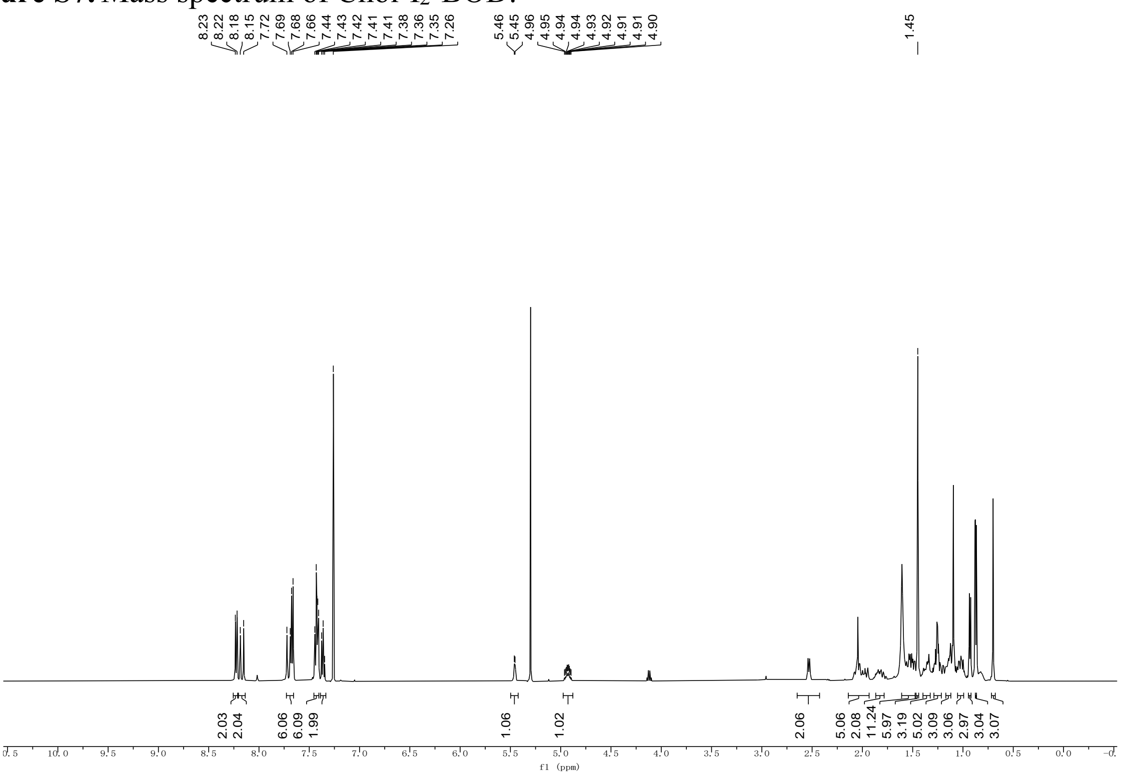

Figure S8. ^{1}H NMR spectrum of BDS ($CDCl_3$, 500 MHz).

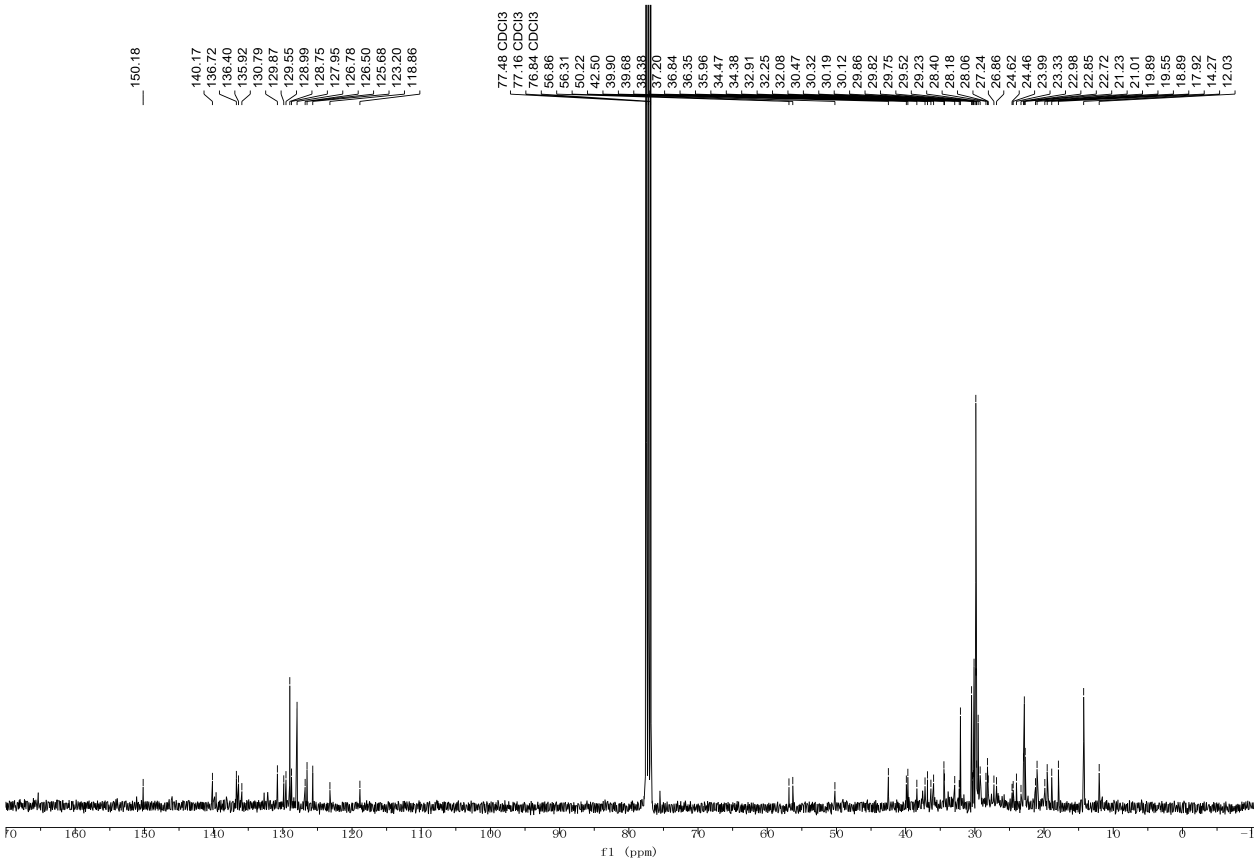

Figure S9. ^{13}C NMR spectrum of BDS ($CDCl_3$, 101 MHz).

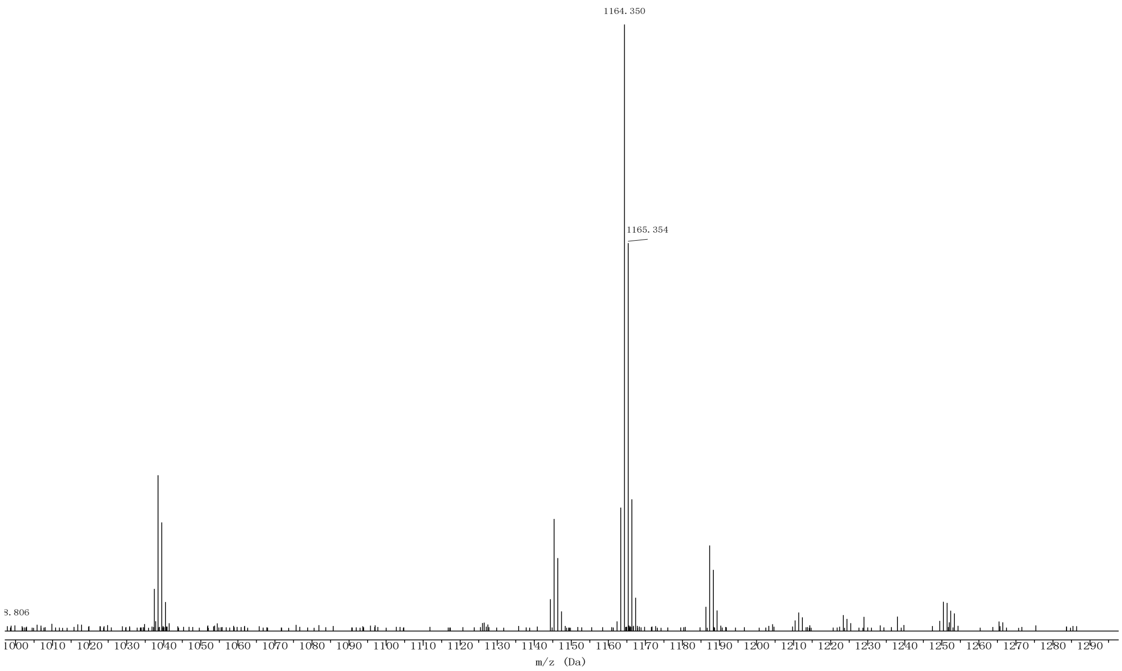

Figure S10. Mass spectrum of BDS.

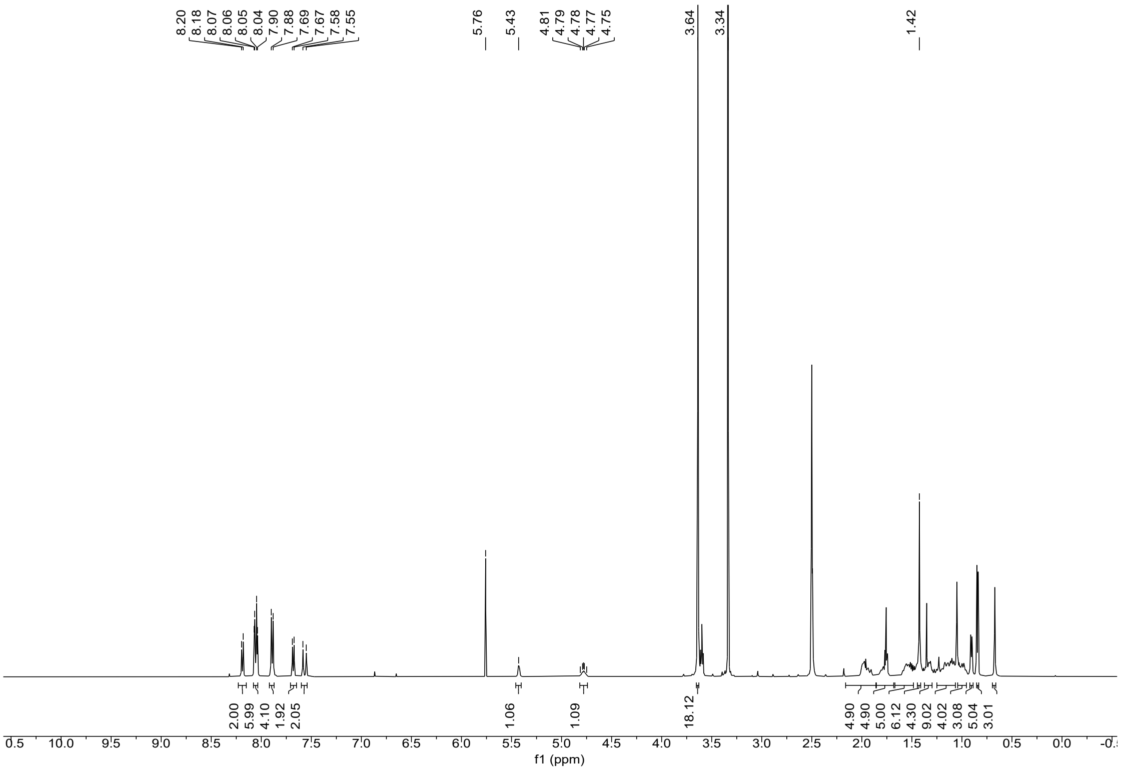

Figure S11. ^{1}H NMR spectrum of BDQ (DMSO-d_6, 500 MHz).

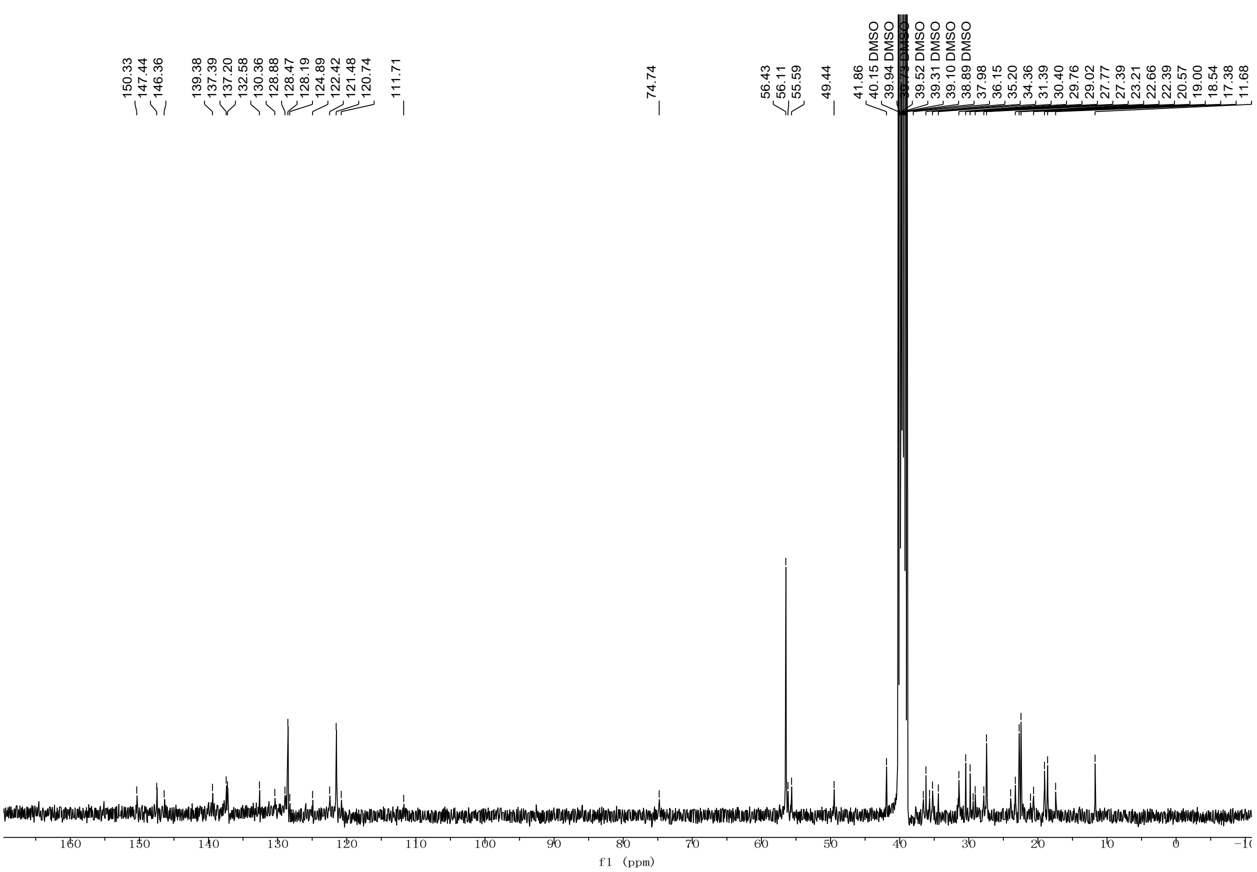

Figure S12. ^{13}C NMR spectrum of BDQ (DMSO-d_6, 101 MHz).

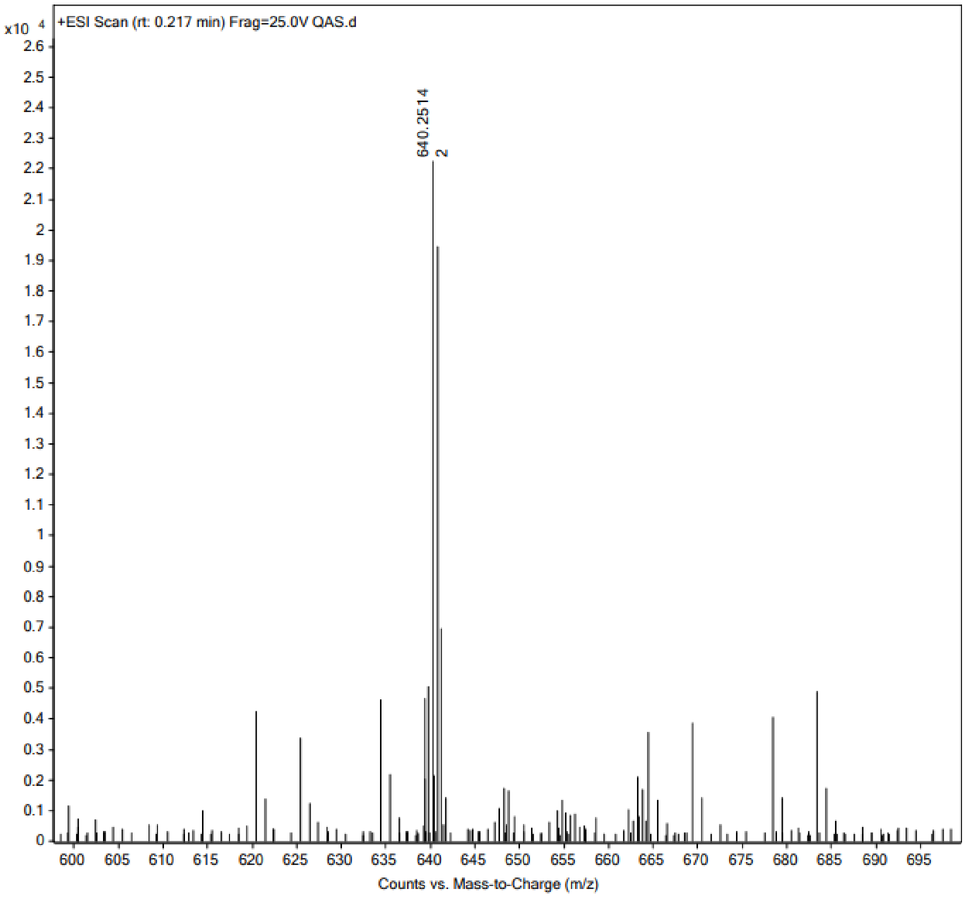

Figure S13. Mass spectrum of BDQ.

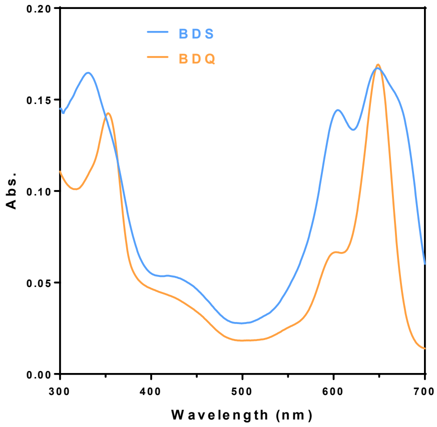

Figure S14. UV-Vis spectra of BDS and BDQ in DMSO/PBS(3:50 v/v).

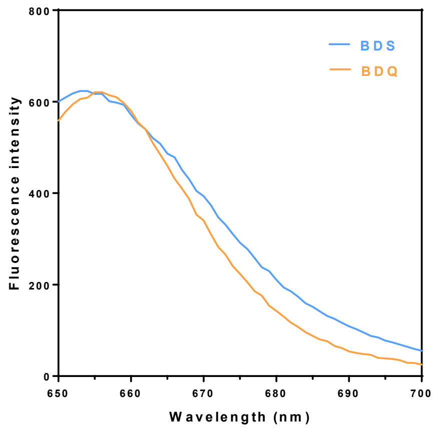

Figure S15. Fluorescence spectra of BDS and BDQ in DMSO/PBS (3:50 v/v) when excited at 640 nm.

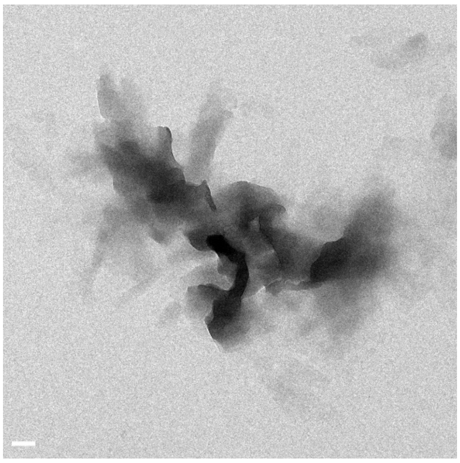

Figure S16. TEM image of BDS-NP. Scale bar = 100 nm.

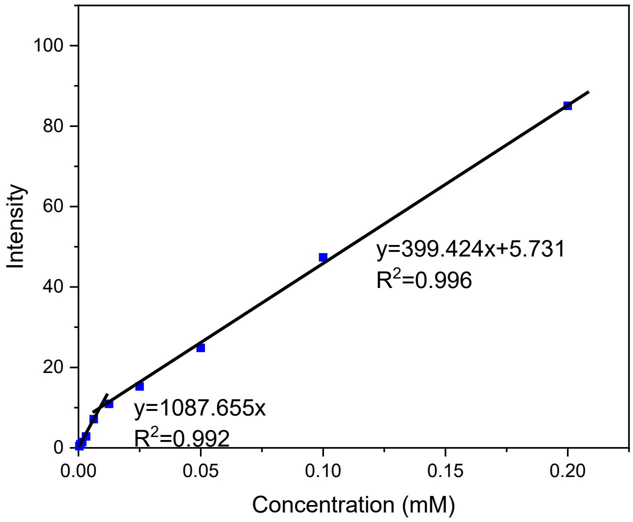

Figure S17. CMC determination of BDQ.

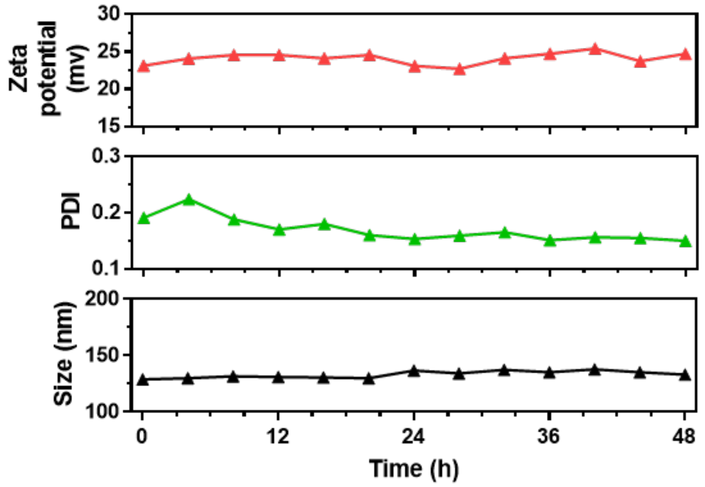

Figure S18. Stability test of BDQ-NP at 25 °C for 48 hours.

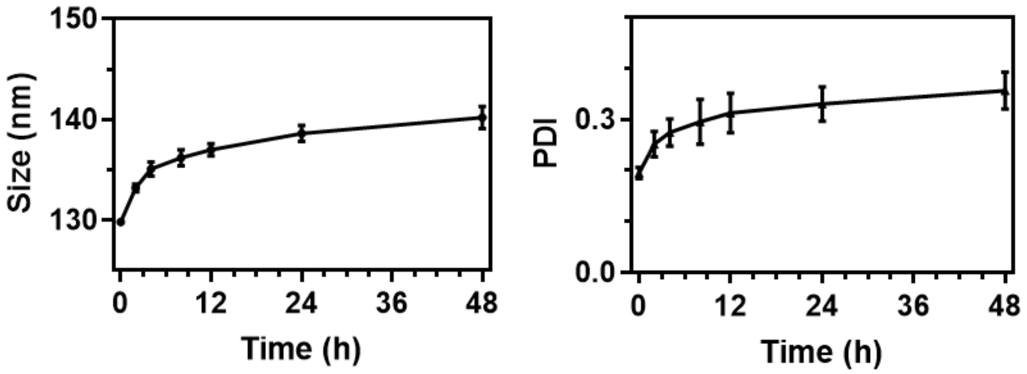

Figure S19. Stability test of BDQ-NP in PBS containing 10% fetal bovine serum over 48 hours.

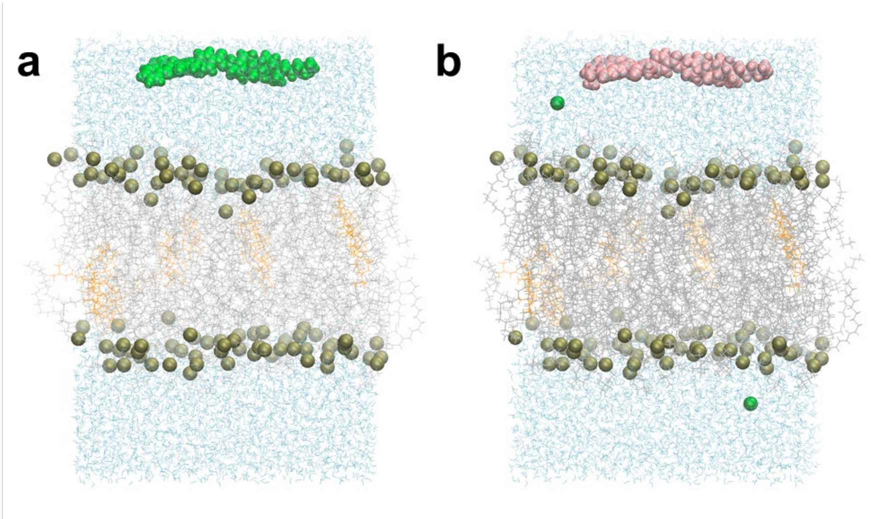

Figure S20. Initial configurations of BDS (a) and BDQ (b) systems after energy minimization. DMPC lipids are shown in gray, phosphorus atoms in tan, cholesterols in orange, BDS in green, BDQ in pink, and water in cyan.

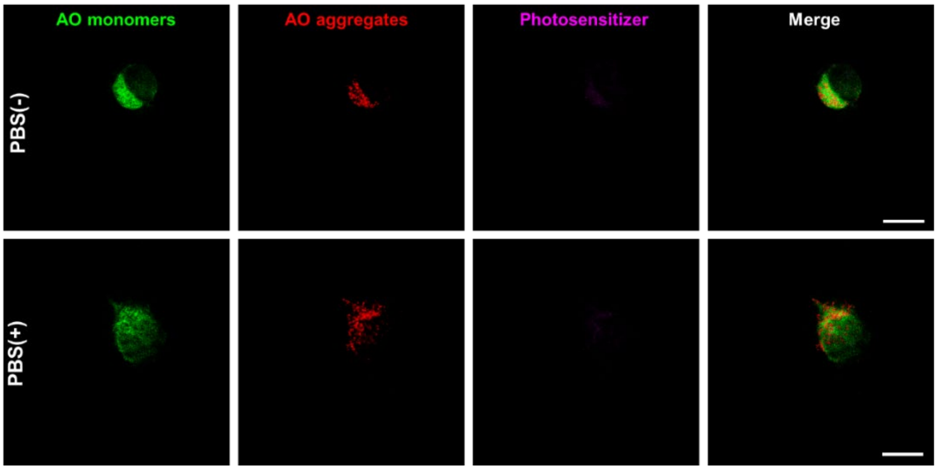

Figure S21. CLSM images of control groups lysosome disruption by AO staining. Scale bar = 20 μm. (+) and (-) represent cells with or without irradiation (660 nm, 60 mW/cm^2, 15 min).

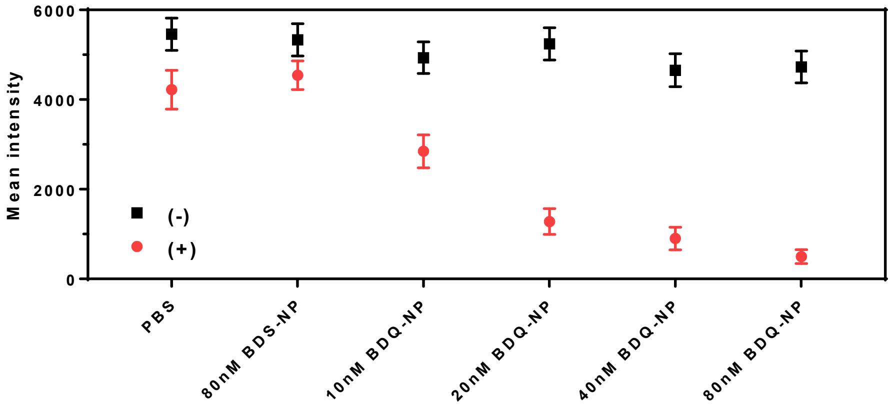

Figure S22. Quantitative analysis of lysosome disruption by flow cytometry with (+) irradiation (660 nm, 60 mW/cm^2, 15 min) or (-) non-irradiation.

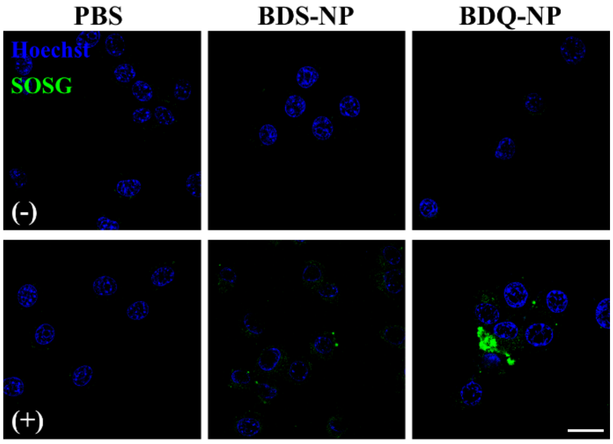

Figure S23. CLSM images of singlet oxygen generation by SOSG assay. Scale bar = 20 μm. (+) and (-) represent cells with or without irradiation (660 nm, 60 mW/cm^2, 15 min).

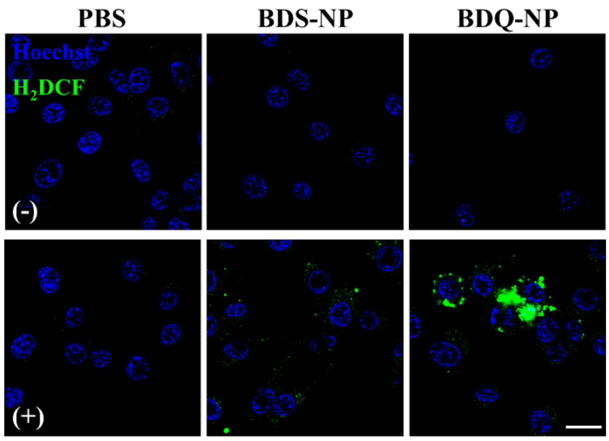

Figure S24. CLSM images of ROS generation by H_2DCF assay. Scale bar = 20 μm. (+) and (-) represent cells with or without irradiation (660 nm, 60 mW/cm^2, 15 min).

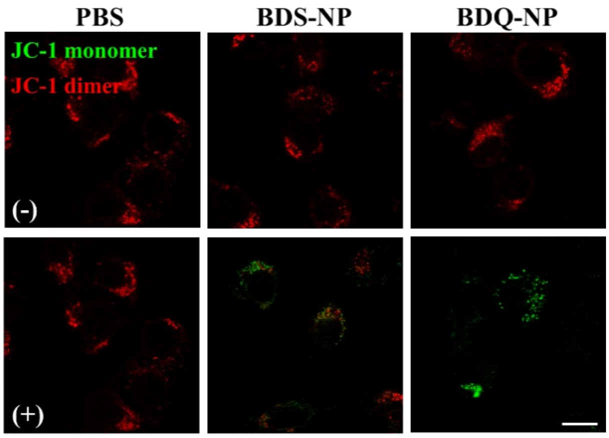

Figure S25. CLSM images of mitochondrial membrane potential loss by JC-1 assay. Scale bar = 20 μm. (+) and (-) represent cells with or without irradiation (660 nm, 60 mW/cm^2, 15 min).

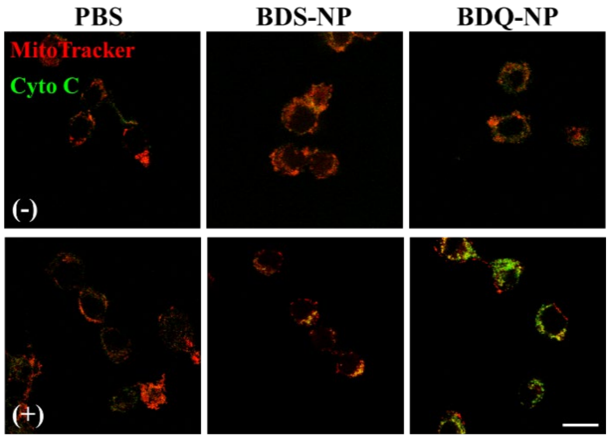

Figure S26. CLSM images of cytochrome c release from mitochondria. Scale bar = 20 μm. (+) and (-) represent cells with or without irradiation (660 nm, 60 mW/cm^2, 15 min).

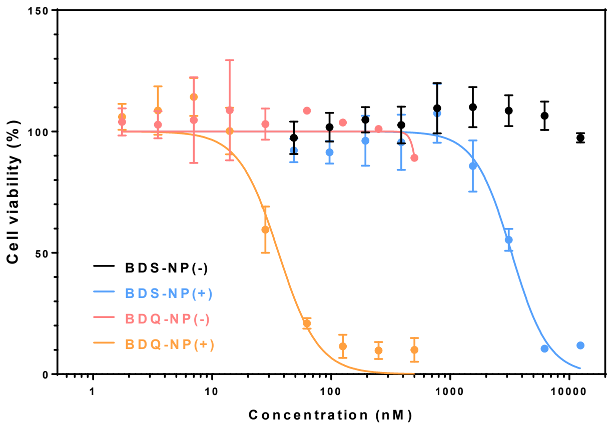

Figure S27. Cytotoxicity by MTS assay. (+) and (-) represent cells with or without irradiation (660 nm, 60 mW/cm^2, 15 min).

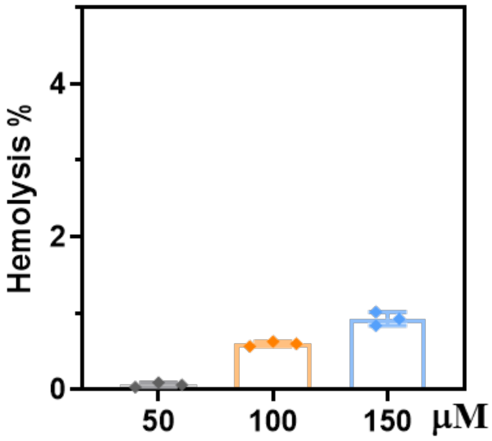

Figure S28. Hemolysis of BDQ-NP.

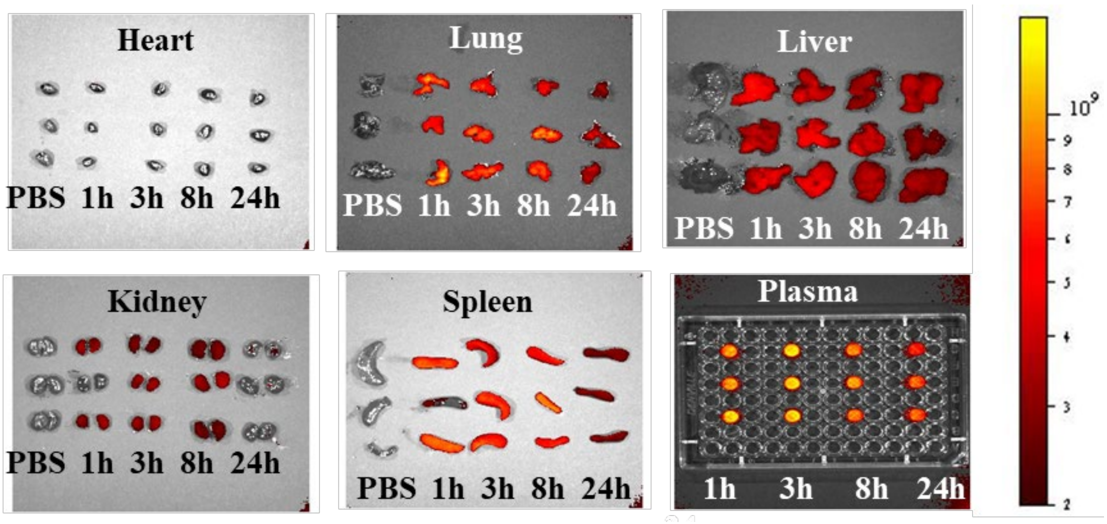

Figure S29. Biodistribution of BDQ-NP in CT26 tumor-bearing mice by IVIS.

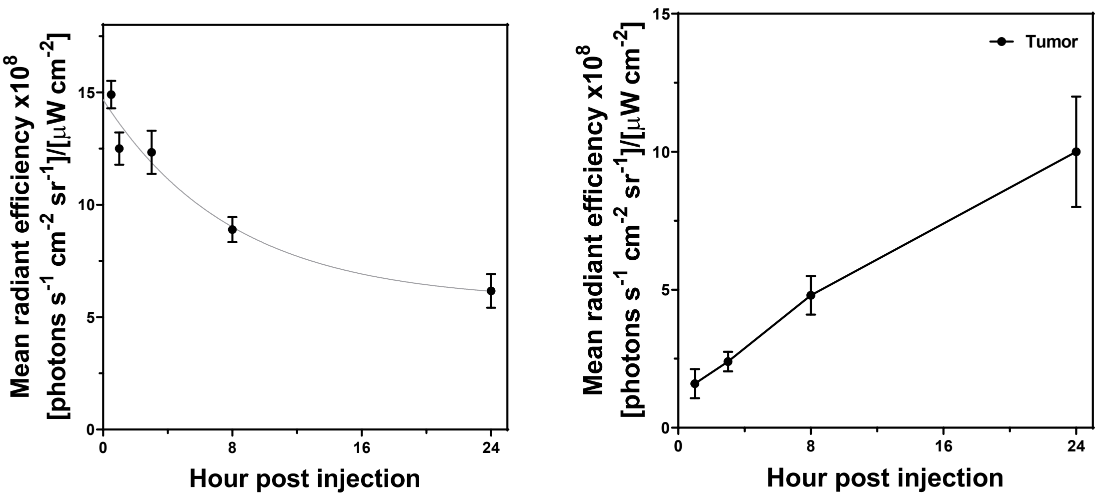

Figure 30. Left, time-dependent BDQ fluorescence intensities in the plasma after i.v. injection of BDQ-NP by IVIS imaging. Right, time-dependent BDQ fluorescence intensities in the tumors of CT26-bearing mice after i.v. injection of BDQ-NP by IVIS imaging.

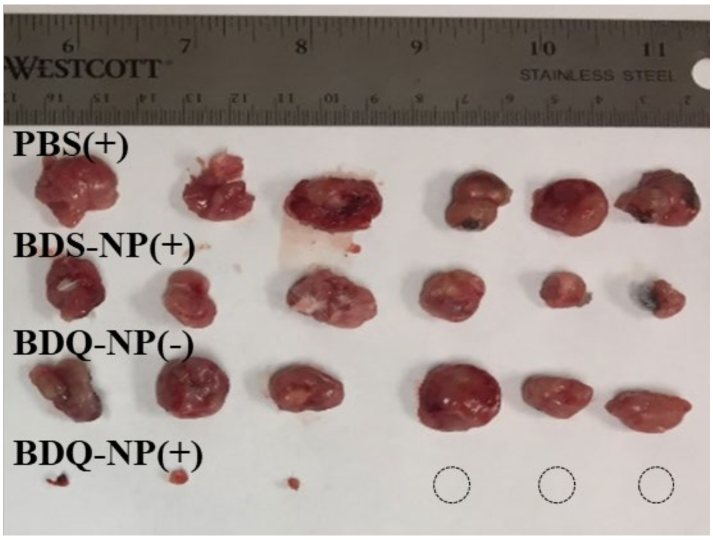

Figure S31. Photo of excised CT26 tumors at the endpoint.

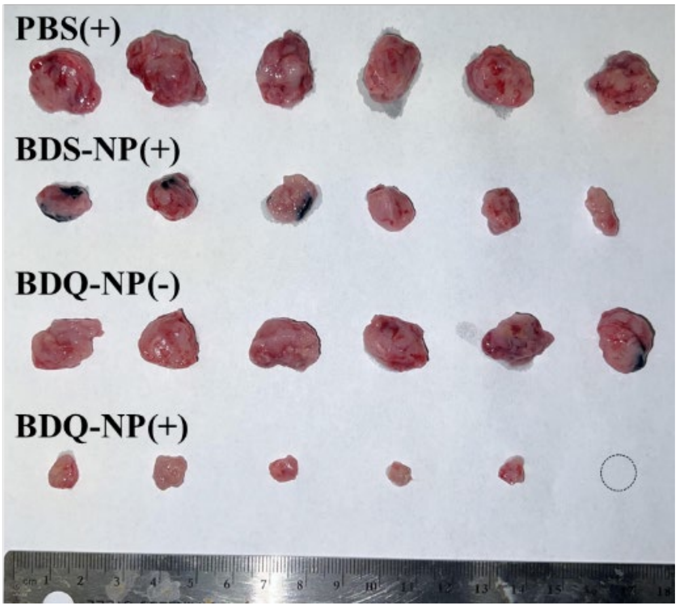

Figure S32. Photo of excised 4T1 tumors at the endpoint.

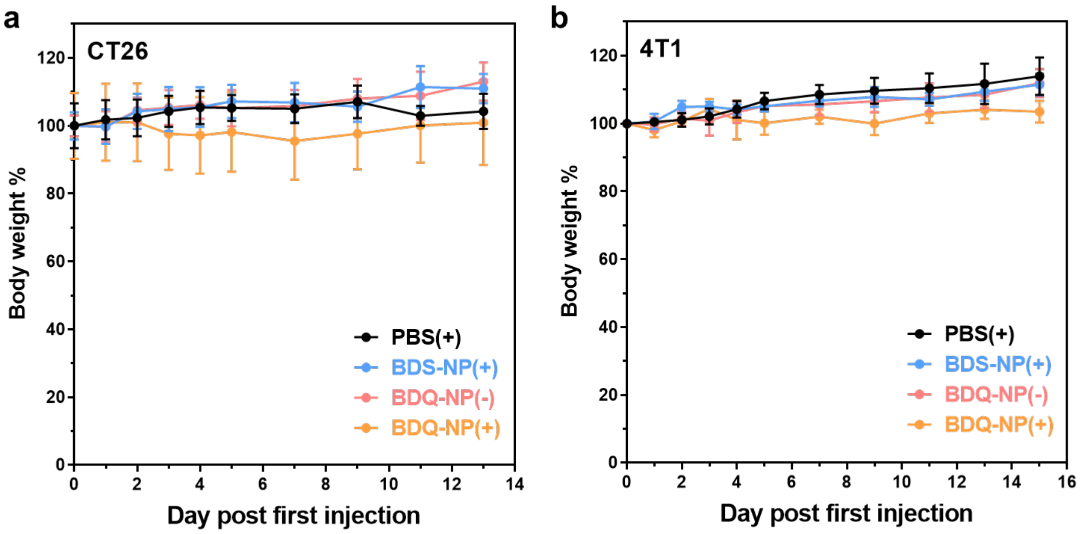

Figure S33. Normalized body weight curves of CT26 (a) or orthotopic 4T1 (b) bearing mice after various treatments (n=6). (+) and (-) represent with or without irradiation (660 nm, 100 mW/cm^2, 15 min).

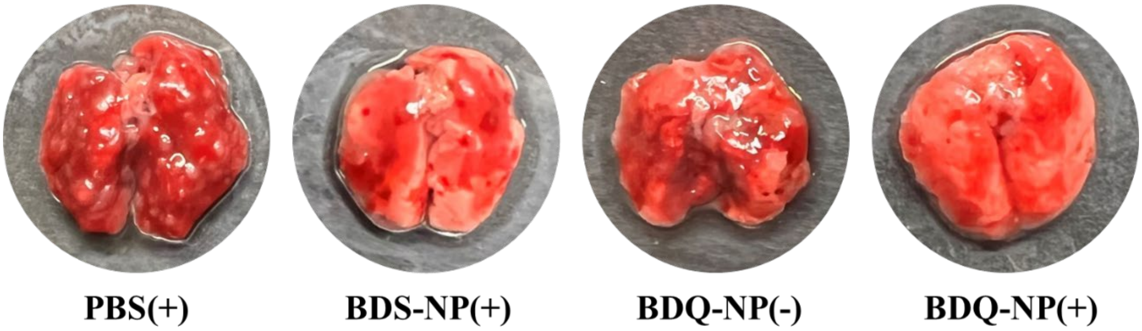

Figure S34. Representative pictures of lungs in different treatment groups. White spots represent tumor nodules. (+) and (-) represent with or without irradiation (660 nm, 100 mW/cm^2, 15 min).

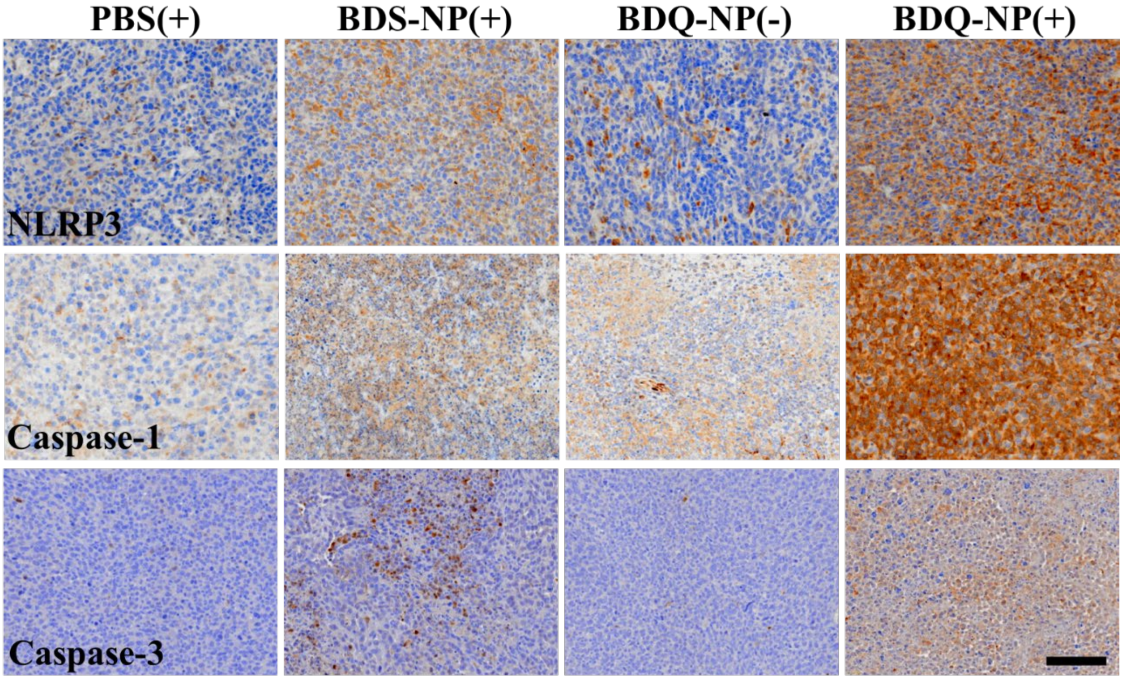

Figure S35. Immunohistochemistry of 4T1 tumor slices after various treatments. Scale bar = 100 μm. (+) and (-) represent with or without irradiation (660 nm, 100 mW/cm^2, 15 min).

Table S1. Number of molecules in the system.

**Molecules**	Cholesterol	DMPC	Water	BDS or BDQ	Cl^-
**Number**	12	116	6400	1	2 (for BDQ)

Table S2. ANOVA analysis and p-values by Turkey test of CT26 tumor volumes on day 13.

Group	Summary	P Value
PBS(+) vs. BDS-NP(+)	*	0.0126
PBS(+) vs. BDQ-NP(-)	ns	0.7828

PBS(+) vs. BDQ-NP(+)	****	<0.0001
BDS-NP(+) vs. BDQ-NP(-)	ns	0.0902
BDS-NP(+) vs. BDQ-NP(+)	**	0.0012
BDQ-NP(-) vs. BDQ-NP(+)	****	<0.0001

Table S3. ANOVA analysis and p-values by Turkey test of 4T1 tumor volumes on day 15.

Group	Summary	P Value
PBS(+) vs. BDS - NP(+)	****	<0.0001
PBS(+) vs. BDQ - NP(-)	ns	0.9969
PBS(+) vs. BDQ - NP (+)	****	<0.0001
BDS - NP(+) vs. BDQ - NP(-)	****	<0.0001
BDS - NP(+) vs. BDQ - NP(+)	****	<0.0001
BDQ - NP(-) vs. BDQ - NP(+)	****	<0.0001

Table S4. ANOVA analysis and p-values by Turkey test of colony numbers formed by metastatic tumor cells on lungs.

Group	Summary	P Value
PBS(+) vs. BDS-NP(+)	**	0.0033
PBS(+) vs. BDQ-NP(-)	ns	0.7111
PBS(+) vs. BDQ-NP(+)	***	0.0002
BDS-NP(+) vs. BDQ-NP(-)	*	0.0127
BDS-NP(+) vs. BDQ-NP(+)	ns	0.0849
BDQ-NP(-) vs. BDQ-NP(+)	***	0.0005

1 S. Guo, H. Zhang, L. Huang, Z. Guo, G. Xiong and J. Zhao, Porous material-immobilized iodo-Bodipy as an efficient photocatalyst for photoredox catalytic organic reaction to prepare pyrrolo [2, 1-a] isoquinoline, *Chemical Communications*, 2013, **49**, 8689-8691.

2 K. W. Dunn, M. M. Kamocka and J. H. McDonald, A practical guide to evaluating colocalization in biological microscopy, *American Journal of Physiology-Cell Physiology*, 2011, **300**, C723-C742.

3 M. Stark, T. F. D. Silva, G. Levin, M. Machuqueiro and Y. G. Assaraf, The Lysosomotropic Activity of Hydrophobic Weak Base Drugs is Mediated via Their Intercalation into the Lysosomal Membrane, *Cells*, 2020, **9**.

4 M. J. Abraham, T. Murtola, R. Schulz, S. Páll, J. C. Smith, B. Hess and E. Lindahl, GROMACS: High performance molecular simulations through multi-level parallelism from laptops to supercomputers, *SoftwareX*, 2015, **1-2**, 19-25.

5 F. Grote and A. P. Lyubartsev, Optimization of Slipids Force Field Parameters Describing Headgroups of Phospholipids, *The Journal of Physical Chemistry B*, 2020, **124**, 8784-8793.

6 A. W. Sousa da Silva and W. F. Vranken, ACPYPE - AnteChamber PYthon Parser interfacE, *BMC Research Notes*, 2012, **5**, 367.

7 U. Essmann, L. Perera, M. L. Berkowitz, T. Darden, H. Lee and L. G. Pedersen, A smooth particle mesh Ewald method, *The Journal of Chemical Physics*, 1995, **103**, 8577-8593.

8 S. Kumar, J. M. Rosenberg, D. Bouzida, R. H. Swendsen and P. A. Kollman, THE weighted histogram analysis method for free-energy calculations on biomolecules. I. The method, *Journal of Computational Chemistry*, 1992, **13**, 1011-1021.

9 W. Humphrey, A. Dalke and K. Schulten, VMD: Visual molecular dynamics, *Journal of Molecular Graphics*, 1996, **14**, 33-38.

10 B. A. Pulaski and S. Ostrand-Rosenberg, Mouse 4T1 Breast Tumor Model, *Current Protocols in Immunology*, 2000, **39**, 20.22.21-20.22.16.
